# The Single-Cell Pediatric Cancer Atlas: Data portal and open-source tools for single-cell transcriptomics of pediatric tumors

**DOI:** 10.1016/j.xgen.2026.101283

**Published:** 2026-06-24

**Authors:** Allegra G. Hawkins, Joshua A. Shapiro, Stephanie J. Spielman, David S. Mejia, Deepashree Venkatesh Prasad, Nozomi Ichihara, Arkadii Yakovets, Avrohom M. Gottlieb, Kurt G. Wheeler, Chante J. Bethell, Steven M. Foltz, Jennifer O’Malley, Casey S. Greene, Jaclyn N. Taroni

**Affiliations:** 1 Childhood Cancer Data Lab, Alex’s Lemonade Stand Foundation, Bala Cynwyd, PA 19004, USA; 2 The University of Texas MD Anderson Cancer Center, UTHealth Houston Graduate School of Biomedical Sciences, Houston, TX 77030, USA; 3 Translational Research, Multiple Myeloma Research Foundation, Norwalk, CT 06851, USA; 4 Center for Health AI, University of Colorado School of Medicine, Aurora, CO 80045, USA; 5 Department of Biomedical Informatics, University of Colorado School of Medicine, Aurora, CO 80045, USA

**Keywords:** single-cell RNA-seq, atlas, pediatric cancer, Nextflow

## Abstract

The Single-Cell Pediatric Cancer Atlas (ScPCA) Portal is a resource for uniformly processed single-cell and single-nucleus RNA sequencing (RNA-seq) data and de-identified metadata from pediatric tumor samples. Originally comprising data from 10 projects funded by Alex’s Lemonade Stand Foundation (ALSF), the Portal currently contains summarized gene expression data for over 700 samples across 55 cancer types from ALSF-funded and community-contributed datasets. Downloads include expression data as SingleCellExperiment or AnnData objects containing raw and normalized counts, principal-component analysis (PCA) and uniform manifold approximation and projection (UMAP) coordinates, automated cell-type annotations, and copy-number variation estimates, along with summary reports. Some samples have additional data from bulk RNA-seq, spatial transcriptomics, and/or feature barcoding (e.g., CITE-seq) included in the download. All data on the Portal were uniformly processed using scpca-nf, an efficient and open-source Nextflow workflow that uses alevin-fry to quantify gene expression. Comprehensive documentation, including file descriptions and a getting-started guide, are available online.

## Highlights

• The Single-Cell Pediatric Cancer Atlas compiles pediatric cancer scRNA-seq data
• Data are uniformly processed via an open-source Nextflow pipeline (scpca-nf)
• Downloads are available in standard formats for R and Python users
• Over 50 pediatric cancer types are represented

## Graphical abstract

**Figure:**
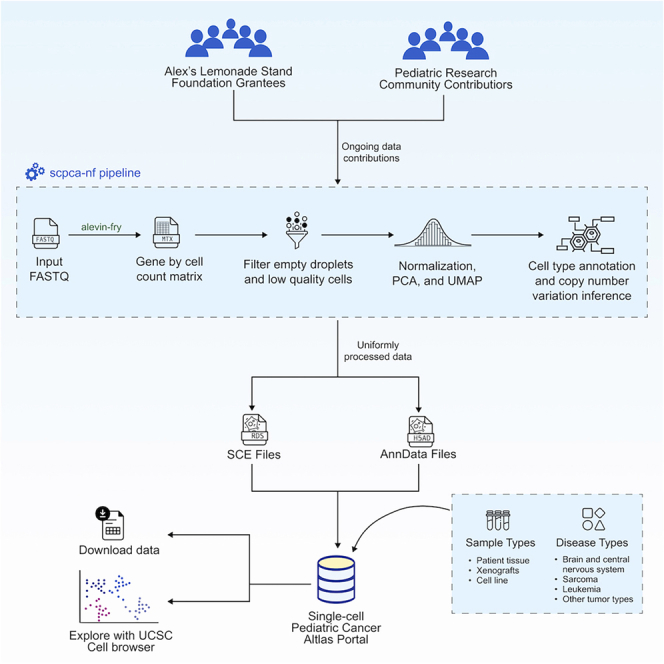

## Teaser

Hawkins et al. introduce the Single-Cell Pediatric Cancer Atlas: a uniformly processed collection of sc/snRNA-seq data from pediatric cancer samples and models. 700 samples from 55 pediatric cancer types can be downloaded in standardized formats for R and Python ecosystems.

## Introduction

The number of studies employing single-cell and single-nucleus RNA sequencing (sc/snRNA-seq) has grown rapidly since their introduction.^1^ Unlike its predecessor, bulk RNA-seq, which averages expression profiles of all cells within a sample, single-cell technology quantifies gene expression in individual cells. Tumors are transcriptionally heterogeneous, highlighting the importance of using sc/snRNA-seq to study tumor samples.^2^ Researchers can use sc/snRNA-seq data from patient tumor samples to analyze and identify individual cell populations that may influence tumor growth, resistance, and metastasis,^3^ as well as how tumor cells interact with normal cells in the tumor microenvironment.^4^

As the number of sc/snRNA-seq datasets expands, efforts have emerged to create centralized data resources. For example, CELLxGENE^5^^,^^6^ offers gene expression data from samples spanning hundreds of cell types in standardized analysis formats. Other resources, such as the Human Cell Atlas (HCA) and the Human Tumor Atlas Network (HTAN), offer harmonized data, enabling reliable cross-sample comparisons and discovery across diverse biological contexts and disease types. The HCA, which provides a comprehensive map of all cell types in the human body using single-cell genomics, contains uniformly processed sc/snRNA-seq data from normal tissue, with few samples derived from diseased tissue.^7^ The HTAN also hosts a collection of genomic data from tumors across multiple cancer types, including sc/snRNA-seq.^8^

Despite these resources, there have been considerably fewer efforts to harmonize and distribute data specifically from pediatric tumors. As pediatric cancer is much less common than adult cancer, fewer samples are available, and access to pediatric tumor data is often limited.^9^ Recently, Xu and colleagues highlighted a lack of standardization of pediatric cancer single-cell data as a barrier to reuse in their attempt to create an atlas.^10^ Thus, it is imperative to make harmonized data from pediatric tumors accessible to researchers.^11^ To address this unmet need, Alex’s Lemonade Stand Foundation (ALSF) and the Childhood Cancer Data Lab developed and maintain the Single-Cell Pediatric Cancer Atlas (ScPCA) Portal (https://scpca.alexslemonade.org/), a data resource for sc/snRNA-seq data of pediatric tumor samples.

The ScPCA Portal holds uniformly processed summarized gene expression from 10× Genomics droplet-based sc/snRNA-seq for over 700 samples from a diverse set of 55 types of pediatric cancers. Originally comprising data from 10 ALSF-funded projects, the Portal has since expanded to include data contributed by pediatric cancer research community members. The Portal also includes data obtained from bulk RNA-seq, spatial transcriptomics, and feature-barcoding methods such as CITE-seq and cell hashing. All data on the Portal are available in formats ready for downstream analysis with common workflow ecosystems, including SingleCellExperiment objects used by R/Bioconductor^12^ or AnnData objects used by scverse and related Python modules.^13^ Downloaded objects contain raw and normalized gene expression counts, dimensionality reduction results, cell-type annotations, and copy-number variation (CNV) estimates. As of May 2026, over 950 unique downloaders had accessed the Portal since its launch.

To ensure uniform processing of all current and future Portal data, we created scpca-nf, an open-source Nextflow^14^ pipeline (https://github.com/AlexsLemonade/scpca-nf). scpca-nf increases transparency and facilitates analyses across multiple samples and projects without re-processing. The scpca-nf workflow uses alevin-fry^15^ for fast and efficient quantification of single-cell gene expression for all samples on the Portal, including sc/snRNA-seq data and any associated CITE-seq or cell hash data. It is also a resource that allows researchers to process their own samples for comparison to Portal data or for submission to the Portal.

Here, we present the ScPCA as a freely available resource for all pediatric cancer researchers. The ScPCA Portal provides downloads ready for immediate use, allowing researchers to skip time-consuming data re-processing and wrangling steps. We provide comprehensive documentation about data processing and the contents of the Portal, including a guide to getting started working with an ScPCA dataset (https://scpca.readthedocs.io/). The ScPCA Portal advances pediatric cancer research by accelerating researchers’ ability to answer important biological questions.

## Results

### The ScPCA Portal

In March 2022, the Childhood Cancer Data Lab launched the ScPCA Portal to make uniformly processed, summarized sc/snRNA-seq data and de-identified metadata from pediatric tumor samples openly available for download by the research community. Data on the Portal were obtained using two different mechanisms: raw data were accepted from ALSF-funded investigators and processed with our open-source pipeline scpca-nf or investigators processed their raw data using scpca-nf and submitted the output for inclusion on the Portal.

All samples on the Portal include a core set of metadata obtained from investigators, including age, sex, diagnosis, subdiagnosis, tissue location, and disease stage. Most include additional metadata, such as treatment or tumor stage, if provided. We standardized all provided metadata to maintain consistency across projects. Where applicable, we include ontology term identifiers, which standardize metadata and facilitate comparisons across Portal and external datasets, along with human-readable values. We use ontology term identifiers obtained from HsapDv (age),^16^ PATO (sex),^17^^,^^18^ NCBI taxonomy (organism),^19^^,^^20^ MONDO (disease),^21^^,^^22^ UBERON (tissue),^23^^,^^24^^,^^25^ and Hancestro (ethnicity, if applicable)^26^^,^^27^ (Table 1).

**Table 1:** Assignment of metadata fields to ontology terms

Metadata field	Ontology term description
Age	ontology term obtained from HsapDv^16^; for ages 0–11 months, the HsapDv for age in months was used; for ages 12 months and greater, the HsapDv for age in years was used
Sex	ontology term obtained from PATO, either male (PATO:0000384), female (PATO:0000383), or unknown^17^^,^^18^
Organism	NCBI taxonomy term for organism; currently, all available samples are from *Homo sapiens* or NCBITaxon:9606^19^^,^^20^
Diagnosis	the most appropriate MONDO term based on the provided diagnosis^21^^,^^22^; an exact match was identified for most samples, but in some cases, the most closely related term was used
Tissue of origin	the most appropriate UBERON term based on the provided tissue of origin^23^^,^^24^^,^^25^; an exact match was identified for most samples, but in some cases, the most closely related term was used
Ethnicity (if applicable)	if the submitter provided ethnicity, the associated Hancestro term^26^^,^^27^; if ethnicity is unavailable, unknown is used

**Figure 1:**
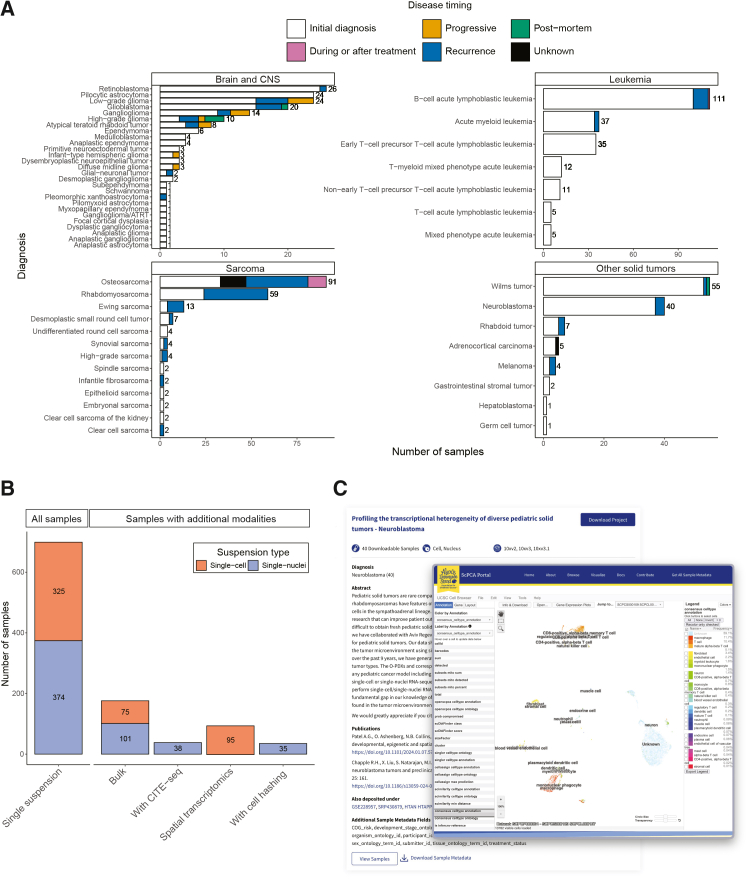
Overview of ScPCA Portal contents (A) Barplots showing sample counts across four main cancer groupings in the ScPCA Portal with the total number of samples per cancer type displayed. Bars are colored by the number of samples with the indicated disease timing. (B) Barplot showing sample counts across modalities present in the ScPCA Portal. All sc/snRNA-seq samples in the Portal are shown under the “all samples” heading, a subset of which also have additional modalities as shown under the “samples with additional modalities” heading. As indicated, sample suspensions are either single cell or single nucleus. For example, 75 single-cell samples and 101 single-nucleus samples have accompanying bulk RNA-seq data. Note that two samples were sequenced with both single-cell and single-nucleus suspensions. Samples with only bulk RNA-seq or spatial transcriptomics modalities are not shown. (C) Example of a project card as displayed on the “browse” page of the ScPCA Portal and a “visualize” view for a library within that project, colored by consensus cell-type annotation. This project card and visualized sample are from project SCPCP000004.^35^^,^^36^ Project cards include information about the sample number, technologies, modalities, additional sample metadata information, submitter-provided diagnoses, and a submitter-provided abstract. Where available, citation information and other databases hosting these data are also provided. The visualization employs the UCSC Cell Browser,^37^ enabling interactive exploration with coloring options for cell types, gene-level expression, and other per-cell annotations created by scpca-nf. See also Table S1.

The Portal contains over 700 samples from 55 tumor types.^28^^,^^29^^,^^30^^,^^31^^,^^32^^,^^33^^,^^34^ Figure 1A summarizes all samples from patient tumors and patient-derived xenografts currently available on the Portal, including the diagnosis and collection time. The most common sample diagnosis on the Portal is leukemia, followed by sarcoma and soft tissue tumors, brain and central nervous system (CNS) tumors, and a variety of other solid tumors. The Portal also contains human tumor cell line samples (*n* = 6) and non-cancerous samples (*n* = 6).

Sample data includes summarized sc/snRNA-seq gene expression data from the 10× Genomics droplet-based platform. Some samples also include additional data, such as CITE-seq quantification of cell-surface protein levels with antibody-derived tags (ADTs; *n* = 95)^38^ or hashtag oligonucleotide (HTO) quantification for multiplexed samples (*n* = 35).^39^ In some cases, multiple libraries from the same sample were created for additional assays, either for bulk RNA-seq (*n* = 182) or spatial transcriptomics (*n* = 41). The Portal does not distribute raw FASTQ files, but we link to raw data sources in external repositories, such as the Database of Genotypes and Phenotypes (dbGaP),^40^^,^^41^ when available. Figure 1B summarizes sequencing modalities (additional details are provided in Table S1).

The Portal organizes samples into projects, defined as collections of similar samples from an investigator. Users can filter projects based on diagnosis, modalities, 10× Genomics kit version, and whether patient-derived xenografts or cell lines are included. The project card displays an abstract, the total number of samples, a list of all present diagnoses, and links to any external information associated with the project, including publications, and links to external resources, e.g., SRA or Gene Expression Omnibus (GEO) (Figure 1C). The project card also indicates sequencing metadata such as the 10× Genomics kit version; the suspension type (cell or nucleus); whether additional sequencing, such as bulk RNA-seq, is present; or whether the samples were multiplexed using cell hashing.

The Portal also provides visualization of individual samples via the UCSC Cell Browser interface,^37^ as shown in Figure 1C. Interactive uniform manifold approximation and projection (UMAP) allows users to explore cells within each sample, coloring by cell-type annotations, gene expression values, or other calculated metrics.

### Uniform processing of data available on the ScPCA Portal

To process data for the Portal, we developed scpca-nf, an open-source and efficient Nextflow^14^ workflow for quantifying sc/snRNA-seq data. Nextflow is a workflow management system that facilitates multi-step and long-running bioinformatics processes in a portable and reproducible manner across computing environments, including high-performance computing clusters and cloud-based computing.^42^ Nextflow allows seamless dependency management by running each process in a specified container image, making the workflow easily portable for general use. Setup requires only installing Nextflow and a supported container engine, managing a configuration file for the computing environment, and organizing input files.

When building scpca-nf, we sought a fast and memory-efficient tool for gene expression quantification to minimize processing costs with comparable performance to the widely used Cell Ranger platform.^43^^,^^44^ Our comparisons between alevin-fry^15^ and Cell Ranger showed that alevin-fry had lower run time and memory usage (Figure S1A) but retained comparable mean gene expression (Figure S1B), total UMIs per cell (Figure S1C), and total genes detected per cell (Figure S1D). We therefore used salmon alevin and alevin-fry^15^ in scpca-nf to quantify gene expression data.

**Figure 2:**
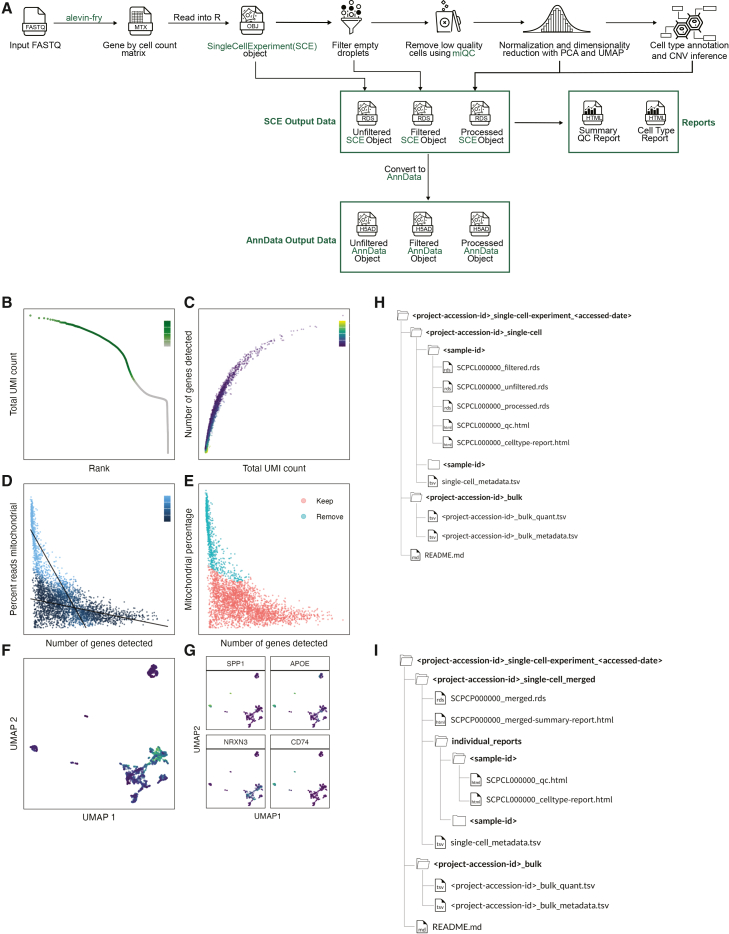
Overview of the scpca-nf workflow and download file structures (A) Overview of scpca-nf, the primary workflow for processing sc/snRNA-seq data for the ScPCA Portal. Mapping is first performed with alevin-fry to generate a gene-by-cell count matrix, which is read into R and converted into a SingleCellExperiment (SCE) object (Unfiltered SCE Object). Next, empty droplets are filtered out (Filtered SCE Object). The filtered object undergoes additional post-processing, including removing low-quality cells, normalizing counts, and performing dimensionality reduction using principal-component analysis and UMAP. The object undergoes cell-type annotation and CNV inference (Processed SCE Object). A summary QC report and a supplemental cell-type report are prepared and exported. Finally, all SCE files are converted to AnnData format and exported. (B–G) Abbreviated versions of figures that appear in the summary QC report, shown here for SCPCL000001,^33^ as follows: (B) the total UMI count for each cell in the Unfiltered SCE Object, ordered by rank. Points are colored by the percentage of cells that pass the empty droplets filter. (C) The number of genes detected in each cell passing the empty-droplet filter against the total UMI count. Points are colored by the percentage of mitochondrial reads in the cell. (D) miQC model diagnostic plot showing the percentage of mitochondrial reads in each cell against the number of genes detected in the Filtered SCE Object. Points are colored by the probability that the cell is compromised as determined by miQC. (E) The percentage of mitochondrial reads in each cell against the number of genes detected in each cell. Points are colored by whether the cell was kept or removed, as determined by both miQC and a minimum unique gene count cutoff, prior to normalization and dimensionality reduction. (F) UMAP of log-normalized RNA expression colored by the number of genes detected. (G) UMAP of log-normalized RNA expression for the top four most variable genes, colored by the given gene’s expression. In the actual summary QC report, the top 12 most highly variable genes are shown. (H) File download structure for an ScPCA Portal project download in SCE format. The download folder is named according to the project ID, data format, and the date it was downloaded. Download folders contain a folder for all single-cell data, _single-cell. Here, each sample ID has a dedicated folder containing the three processing levels of the expression data, the summary QC report, and the cell-type report, all named according to the ScPCA library ID. The single-cell_metadata.tsv file contains sample metadata for all samples included in the download. The README.md file provides information about the contents of each download file, additional contact and citation information, and terms of use for data downloaded from the ScPCA Portal. The folder _bulk—only present for projects with bulk RNA-seq data—contains a gene-by-sample matrix of counts quantified by salmon and associated metadata for samples with bulk RNA-seq data. (I) File download structure for an ScPCA Portal merged project download in SCE format. As in (H), the download folder is named by project ID, format, and date. Download folders contain a folder for all single-cell data, _single-cell_merged, with a single merged object containing all samples in the given project and a summary report detailing the merged object’s contents. Summary QC and cell-type reports for each library are provided in the individual_reports folder arranged by their library ID. As in (H), additional files single-cell_metadata.tsv, _bulk_quant.tsv, _bulk_metadata.tsv, and README.md are also included. See also Figures S1–S3.

Taking FASTQ files as input, scpca-nf aligns reads using the selective alignment option in salmon alevin to an index with transcripts corresponding to spliced cDNA and intronic regions, i.e., the splici index in alevin-fry (Figure 2A). alevin-fry outputs a gene-by-cell count matrix for all barcodes identified, even those that may not contain true cells.

scpca-nf filters empty droplets and low-quality cells and performs normalization, dimensionality reduction, cell-type annotation, and CNV inference (Figure 2A). We used the Bioconductor ecosystem^45^^,^^46^ for filtering, normalization, and dimensionality reduction because of its rich documentation, community adoption, and relatively small file sizes. Unfiltered gene-by-cell count matrices are filtered to remove barcodes that are unlikely to contain cells using DropletUtils::emptyDropsCellRanger().^47^^,^^48^ Low-quality cells are identified and removed with miQC,^49^ which jointly models the proportion of per-cell mitochondrial reads and detected genes and calculates a probability that each cell is compromised. The remaining cell counts are normalized,^50^ and dimensionality reduction representations are calculated using both principal-component analysis (PCA) and UMAP.^51^ Cell types are classified using three automated methods, SingleR,^52^ CellAssign,^53^ and SCimilarity,^54^ and a consensus cell-type label is derived from these labels. Finally, CNV is estimated for each cell with inferCNV.^55^

To support both R and Python users, downloads are available as either SingleCellExperiment or AnnData^56^ objects. The workflow outputs three different SingleCellExperiment objects to RDS files: a processed object containing dimensionality reduction results, cell-type annotations, and CNV inference; an unfiltered object with no processing; and a filtered object with the empty-droplet-filtered gene-by-cell matrices. scpca-nf then converts all SingleCellExperiment objects to AnnData objects (H5AD files; Figure 2A). Downloads contain the unfiltered, filtered, and processed objects from scpca-nf, allowing users to either perform custom filtering and normalization or start their analysis from a processed object. Providing unfiltered raw counts is consistent with the recommendations in Xu et al.^10^ for maximizing reusability when sharing pediatric cancer single-cell data.

All Portal downloads include a quality control (QC) report with a summary of processing information (e.g., alevin-fry version), library statistics (e.g., the total number of cells), and diagnostic plots for each library (Figures 2B–2G). A knee plot displaying total UMI counts across all (including empty) droplets indicates the effects of empty-droplet filtering (Figure 2B). For each cell remaining after filtering, the total UMI count, genes detected, and mitochondrial fraction are presented (Figure 2C). We include plots showing the miQC model and the results of miQC filtering (Figures 2D and 2E). We also provide a UMAP with cells colored by the number of genes detected and a faceted UMAP plot colored by the expression of highly variable genes (Figures 2F and 2G).

### Processing samples with additional modalities

scpca-nf includes modules for processing additional sequencing modalities, including CITE-seq (ADT),^38^ multiplexed (cell hashing),^39^ spatial transcriptomics, or bulk RNA-seq.

For CITE-seq libraries, ADT reads are quantified using salmon alevin and alevin-fry (Figure S2A). The workflow performs ADT-by-cell counts matrix normalization (see STAR Methods for details) and calculates QC statistics that users can employ for additional filtering. Associated QC reports include additional ADT-related statistics and ADT-specific diagnostic and exploratory plots (Figures S2B–S2D).

For multiplexed libraries, the HTO FASTQ files are quantified using salmon alevin and alevin-fry (Figure S2E). Although scpca-nf quantifies the HTO data and includes an HTO-by-cell counts matrix in all objects, final demultiplexing is not performed. Instead, scpca-nf applies multiple demultiplexing methods, including demultiplexing with DropletUtils::hashedDrops(),^47^^,^^48^ Seurat::HTODemux(),^39^ and genetic demultiplexing^57^ if bulk RNA-seq data from constituent samples are available. All demultiplexing results are saved in the filtered and processed SingleCellExperiment objects, and HTO-specific library statistics are included in the QC report.

For bulk RNA-seq data, scpca-nf trims reads using fastp,^58^ quantifies expression with salmon (Figure S3A),^59^ and outputs the gene-by-sample counts matrix for all samples in a given ScPCA project to a TSV file.

Spatial transcriptomics data are processed with Space Ranger^60^ to quantify expression and process slide images (Figure S3B). The output includes the spot-by-gene matrix along with a summary report produced by Space Ranger.

### Merged objects

Combining data from multiple samples into a single object facilitates joint gene-level analyses, such as differential expression or gene set enrichment analyses. Therefore, we provide a merged object for each ScPCA project containing all raw and normalized gene expression data and metadata for all sc/snRNA-seq libraries (with exceptions described in STAR Methods) produced using our merge.nf workflow (Figure S3C), as well as a merge summary report (Figure S3D). Merged objects are not batch corrected or integrated; users can perform their own batch correction or integration as needed.

### Downloading projects from the ScPCA Portal

Users can download data from individual samples or all data from an ScPCA project as either SingleCellExperiment (RDS) or AnnData (H5AD) objects. When downloading a complete project, users can either download individual files for each sample (Figure 2H) or one file containing the gene expression data and metadata for all project samples as a merged object (Figure 2I). Users can also generate custom datasets by selecting specific samples across projects for a single download. In addition to the web interface, we provide an R package, ScPCAr, for programmatic access, available at https://alexslemonade.github.io/ScPCAr/.

### Annotating cell types

**Figure 3:**
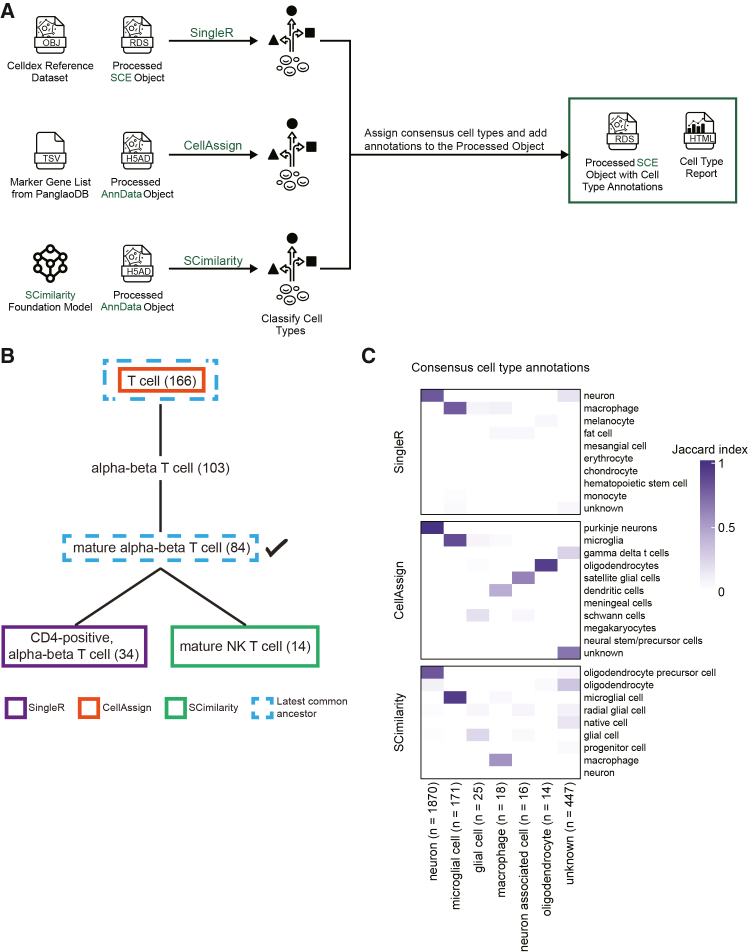
Consensus cell-type annotation in scpca-nf (A) Expanded view of cell-type annotation process within scpca-nf, as introduced in Figure 2A. Cell-type annotation is performed on the Processed SCE Object. SingleR^52^ annotation uses a celldex^52^ reference dataset with ontology labels, CellAssign^53^ annotation uses a list of marker genes compiled from PanglaoDB,^61^ and SCimilarity^54^ annotation uses the SCimilarity foundation model. These cell-typing results, along with consensus cell-type annotations, are then added to the Processed SCE Object. A cell-type summary report that includes information about reference sources, comparisons among cell-type annotation methods, and diagnostic plots is created. Note that cell-type annotations are also included in the Processed AnnData Object (Figure 2A). (B) Diagram of ontology-aware consensus cell-type annotation performed in scpca-nf, using T cell annotation as an example. The numbers in parentheses indicate the number of descendants for each cell type in the Cell Ontology. Pairwise comparisons are made among annotations, and the consensus cell type is the latest common ancestor in the Cell Ontology with the fewest descendants, indicated by the black check mark. (C) Example heatmap (SCPCL000001) as shown in the cell-type summary report comparing the consensus cell-type annotations to automated annotations assigned by SingleR, CellAssign, and SCimilarity. Heatmap cells are colored by the Jaccard similarity index. A value of 1 indicates complete overlap and 0 indicates no overlap between cells annotated with each label. This figure shows only the top seven consensus cell-type annotations with at least three cells, but the heatmap in the cell-type summary report shows all cell types. See also Figure S4 and Table S2.

Cell-type annotation requires knowledge of expected cell types and their associated gene expression patterns, which may be available from public databases or individual publications. Automated annotation methods leveraging public databases are an excellent initial step in this process, as they can be applied consistently and transparently across samples. We therefore include cell-type annotations from three different automated methods, SingleR,^52^ CellAssign,^53^ and SCimilarity,^54^ in all processed SingleCellExperiment and AnnData objects (Figure 3A) (see STAR Methods for details). A cell-type report that includes information about references, comparisons among cell-type annotation methods, and diagnostic plots is also provided.

Some ScPCA projects also have curated cell-type annotations, including tumor cells and disease-specific cell states, provided by submitters. These submitter-provided annotations are in all SingleCellExperiment and AnnData objects (unfiltered, filtered, and processed). These projects’ cell-type reports include additional tables and plots with these annotations, including comparisons to automated cell-typing results.

### Assigning consensus cell types

SingleR, CellAssign, and SCimilarity use different references and approaches. Additionally, most publicly annotated reference datasets compatible with SingleR and CellAssign, including those used here, are derived from normal tissue, making annotating tumor datasets particularly difficult. As consistent cell-type annotations across methods can indicate higher confidence, we used an ontology-aware approach to assign consensus cell-type labels based on the methods’ agreement.

scpca-nf assigns consensus cell types when two of the three automated methods agree. Specifically, pairwise comparisons among automated cell-type annotations identify the latest common ancestor (LCA) in the Cell Ontology.^62^^,^^63^^,^^64^ The consensus cell type is the LCA term with the fewest descendants (Figure 3B). To ensure specificity, cells are only assigned a consensus cell type if the identified LCA has fewer than 170 descendant terms (see STAR Methods for exceptions). This threshold excludes overly general Cell Ontology terms, such as lymphocyte, while retaining meaningful classifications, such as T cell and B cell. Consensus cell types are available in all processed objects. Figure 3C shows an example heatmap comparing automated and consensus cell-type labels for a glioma library on the Portal.

This ontology-based approach accounts for different levels of granularity in annotation references. For example, Figure S4A displays cells annotated as different T cell subtypes by each automated method. Harmonizing annotations into a consensus cell type provides a single, consistent label for each cell (Figure S4B) and facilitates downstream analyses across multiple samples and integration with CNV estimates (Figure S4C).

**Figure 4:**
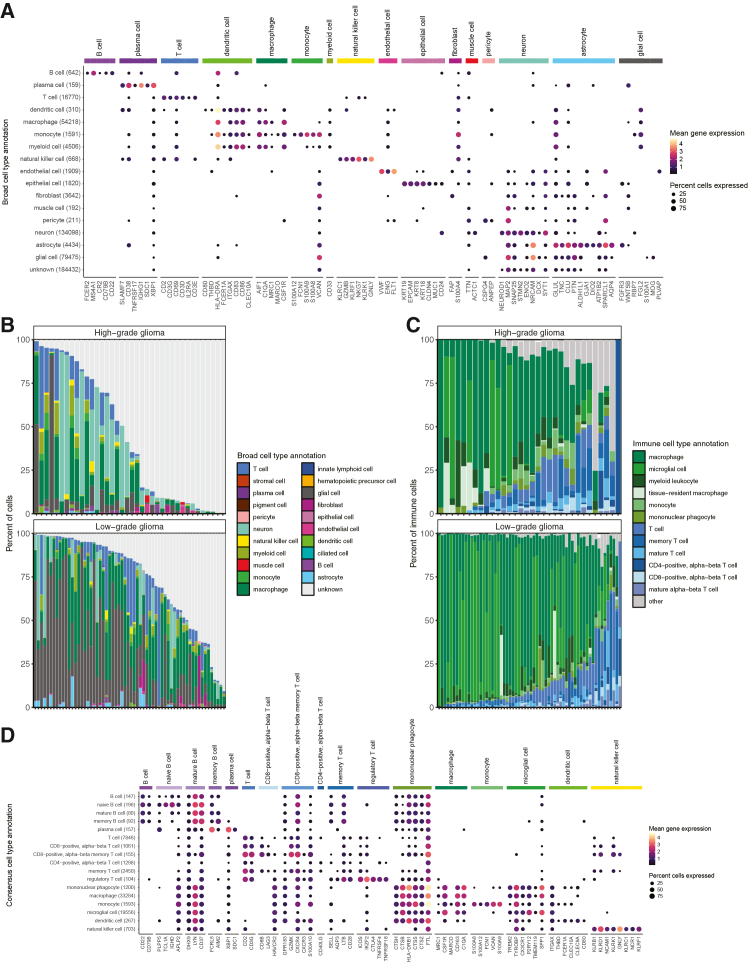
Consensus cell-type annotations in brain and CNS tumors (A) Dot plot showing expression of cell-type-specific marker genes across all libraries from brain and central nervous system (CNS) tumors, excluding multiplexed libraries. Expression is shown for each broad cell-type annotation, each of which represents a collection of similar consensus cell-type annotations. The *y* axis displays broad consensus cell types. The *x* axis displays marker genes, determined by CellMarker 2.0,^65^ used to validate cell types in the top annotation bar. Dots are colored by mean gene expression across libraries and sized proportionally to the percentage of libraries in which they are observed, among all cells with the same broad cell-type annotation in brain and CNS tumor libraries. Up to 10 marker genes are shown per broad cell type. Only broad cell-type annotations present in at least 50 cells across samples in the given diagnosis group are shown. (B) Barplot showing the percentage of each broad consensus cell-type annotation across brain and CNS tumor libraries, separated into high-grade glioma (top) and low-grade glial/glioneuronal (bottom) diagnoses for non-multiplexed libraries from patient tissue samples. (C) Barplot showing all consensus cell types classified as immune cells across brain and CNS tumor libraries, separated into high-grade glioma (top) and low-grade glial/glioneuronal (bottom) diagnoses for non-multiplexed libraries. The percentage of immune cells classified as the indicated consensus cell type is shown. Only libraries comprising at least 1% of immune cells, based on consensus cell-type annotations, are shown. Specific consensus cell types for myeloid and lymphocyte immune cells are shown, with all other consensus immune cell types included in “other.” Myeloid or lymphocyte immune cell types with fewer than 1,000 cells across all libraries are included in “other.” (D) Dot plot as in (A) but restricted to immune cells across all non-multiplexed libraries from brain and CNS tumors and showing consensus rather than broad cell types showing expression of cell-type-specific marker genes, considering only immune cells. Only broad cell-type annotations present in at least 50 cells across samples in the given diagnosis group are shown. Cell types without associated marker genes in CellMarker 2.0 are excluded, including lymphocyte of B lineage, mature T cell, mature alpha-beta T cell, alpha-beta T cell, myeloid leukocyte, and tissue-resident macrophage. See also Figures S5 and S6.

We validated consensus cell types by evaluating cell-type-specific marker gene expression across all cells (Figures 4A and S5), observing high concordance between consensus labels and gene expression. Library-specific versions of Figures 4A and 3C are included in the QC reports.

### Consensus cell-type annotations in brain and CNS samples available on the Portal

Consensus annotations are particularly useful for cross-project comparisons. Figure 4B, for example, displays cell types across all high-grade (HGG) and low-grade (LGG) glial/glioneuronal samples, which originate from six projects and four investigators, and reveals similar cell types across all samples (see Figure S6 for consensus labels across all other samples).

The glioma immune microenvironment is dominated by myeloid cells, including microglia and glioma-associated macrophages, with smaller proportions of lymphocytes such as T cells.^66^^,^^67^ While we observe that most immune cells in glioma samples are myeloid or T cell types, there is notable heterogeneity within HGG and LGG subtypes (Figure 4C). Figure 4D shows cell-type-specific marker expression for more granular immune cell types, which is concordant with the assignments.

### Augmenting cell-type annotations for malignant cell identification

Because the SingleR and CellAssign references contain only normal cells, consensus annotations cannot robustly identify malignant cells. We therefore sought complementary avenues to help identify malignant cells.

**Figure 5:**
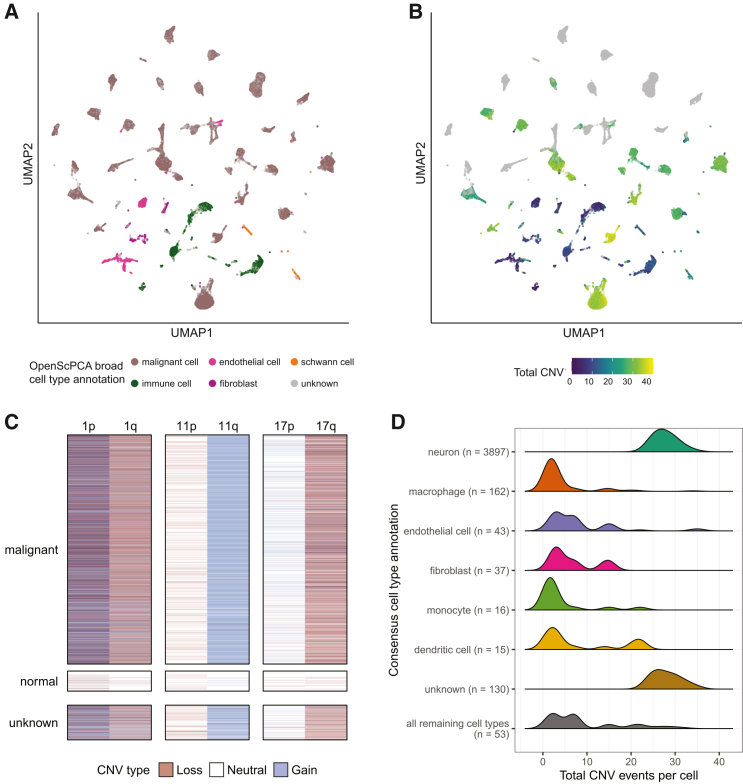
Cell-type annotation and CNV inference on neuroblastoma samples (A and B) UMAP of all libraries in the neuroblastoma-only ScPCA Project SCPCP000004 (*n* = 42). The UMAP was constructed from the merged SCPCP000004 object with equal library weighting, but no batch correction was performed. (A) highlights cell-type annotations made with the OpenScPCA Project, collapsed into broad annotation groups. (B) highlights total per-cell CNV events calculated as the sum of chromosome arms with a CNV event, as estimated by the i6 HMM in InferCNV.^55^ Gray cells in (B) represent libraries excluded from InferCNV inference due to insufficient normal cells for defining the InferCNV reference baseline. (C) Heatmap displaying per-cell CNV events across chromosomes with canonical neuroblastoma alterations^69^^,^^70^^,^^71^ for a single library, SCPCL000130. Each cell in SCPCL000130 is represented by two adjacent rows, the first indicating copy-number gain and the second indicating copy-number loss. The heatmap is grouped by chromosome arm and OpenScPCA Project cell-type annotation, where “normal” cells comprise all non-malignant cells. (D) Ridge plot from the summary QC report (SCPCL000130) of per-cell total CNV distributions across the top seven consensus cell-type annotations. Other consensus cell types are shown in the “all remaining cell types” category.

To this end, we launched the OpenScPCA project,^68^ an open-science collaborative initiative to characterize and analyze Portal data, focusing first on improving cell-type annotations. Thus far, the Portal contains OpenScPCA cell-type annotations for two projects, SCPCP000004 (neuroblastoma) and SCPCP000015 (Ewing sarcoma). Figure 5A displays a UMAP of all libraries in SCPCP000004 highlighting this project’s OpenScPCA annotations, derived using the NBAtlas reference dataset.^69^ Unlike the consensus labels, the OpenScPCA annotations distinguish between normal and malignant cells and contain far fewer uncharacterized cells. Summary cell-type reports for projects with OpenScPCA annotations also include comparisons to scpca-nf annotations.

In addition, scpca-nf applies inferCNV^55^ to estimate copy-number alterations (Figure 2A) for libraries with enough normal cells for a reference (see STAR Methods for details). The CNV estimates complement the consensus cell types by providing a proxy for a cell’s malignant status; cells with high levels of CNVs are more likely to be tumor cells. Across libraries within SCPCP000004, malignant cell-type annotations from OpenScPCA (which does not use CNV information) (Figure 5A) and the total per-cell CNV (Figure 5B) broadly correspond. Figure 5C shows an example neuroblastoma library with canonical neuroblastoma CNV events, including 1q loss, 11q gain, and 17p loss^69^^,^^70^^,^^71^ within malignant cells (Figure 5C). By contrast, normal cells show fewer CNV events. Unknown cells show CNV event signatures similar to malignant cells, suggesting many of them may be malignant.

Malignancy can also be assessed by interpreting consensus labels alongside CNV inferences, which is particularly useful for projects that do not yet have OpenScPCA annotations. Figure 5D shows per-cell CNV distributions for the most common consensus labels in a neuroblastoma library. Unknown and neuron cells have distinctly higher values, suggesting possible malignancy. We see similar patterns in a ganglioglioma library (Figures S4B and S4C), where consensus immune cell types have low CNV values, while other cell types, including oligodendrocyte precursor cells, neuron-associated cells, and unknown cells, have much higher CNV values.

### Analysis of bulk RNA-seq

**Figure 6:**
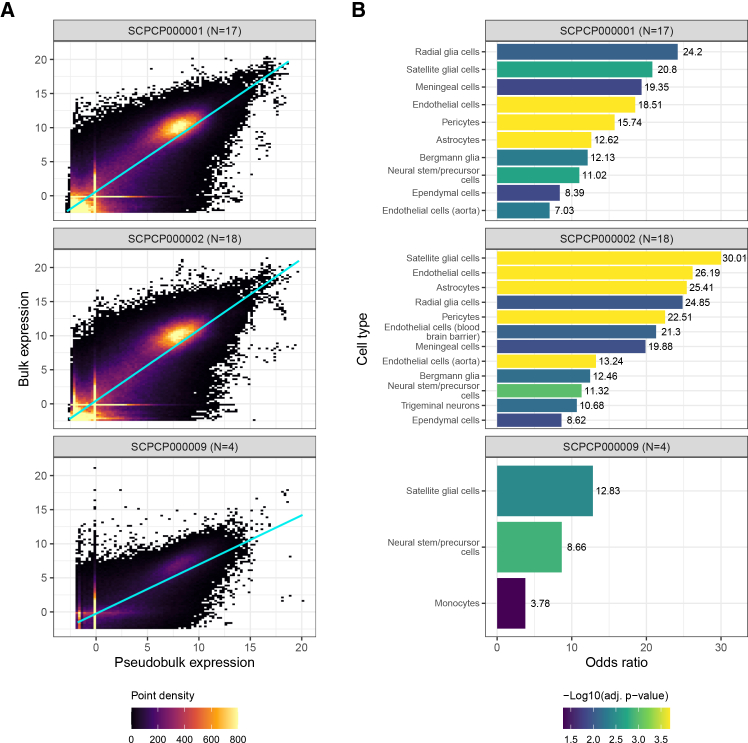
Comparison of bulk and pseudobulk modalities (A) Scatterplots colored by point density of DESeq2-transformed and normalized bulk RNA-seq expression compared to pseudobulk expression from single-cell/nucleus RNA-seq, with a regression line shown. Samples with RNA-seq for both bulk and single-cell/nucleus modalities, excluding multiplexed samples, from ScPCA projects of brain and CNS tumors are shown, with sample counts in parentheses. (B) Odds ratios, which indicate overrepresentation of cell-type marker genes in bulk relative to single-cell/nucleus RNA-seq, from overrepresentation analysis for the same samples shown in (A), colored by false discovery rate (FDR)-corrected significance. 68 cell types were evaluated for each project. *p* values were calculated via permutation testing with 10,000 replicates.-values were calculated via permutation testing with 10,000 replicates. See also Figure S7.

Several projects in the ScPCA Portal contain bulk RNA-seq data. Compared with bulk RNA-seq, sc/snRNA-seq technologies may fail to capture certain cell types^72^ due to technical aspects of library preparation. We therefore asked whether biological signals differed between these two modalities in ways that suggest distinct cell-type distributions. We analyzed ScPCA solid tumors projects with matched modalities, comprising 97 samples across five projects: SCPCP00001 (high-grade glioma), SCPCP000002 (low-grade glioma), SCPCP000006 (Wilms tumor), SCPCP000009 (CNS tumors), and SCPCP000017 (osteosarcoma). As described in STAR Methods, we derived pseudobulk expression matrices for each single-cell or single-nucleus library and compared the pseudobulk and bulk expression using one linear model per project with a random effect controlling for sample (Figures 6A and S7A). As expected, all projects showed a positive relationship between bulk and pseudobulk expression.

We next performed an overrepresentation analysis to probe for differences in gene expression suggestive of different cell-type compositions and/or abundances between modalities, as described in STAR Methods. Several cell-type marker gene sets had higher, but never lower, bulk RNA-seq expression than expected (Figures 6B and S7B).

In brain and CNS tumors, the overrepresented marker gene sets in bulk RNA-seq were primarily stromal and neuronal cell types, all of which are prevalent in glioma tumor microenvironments^73^^,^^74^ (Figure 6B). By contrast, only monocytes and neuronal cell types were overrepresented in the SCPCP000009 bulk RNA-seq data. As SCPCP000009 was sequenced at the single-nucleus level and SCPCP000001 and SCPCP000002 were sequenced at the single-cell level, this difference may reflect reduced immune cell detection sensitivity in single-nucleus approaches.^75^ The other single-nucleus projects similarly showed immune cell overrepresentation in bulk RNA-seq: monocytes for SCPCP000006 and a mix of immune and non-immune cell types for SCPCP000017 (Figure S7B), the latter possibly reflecting challenges in dissociating bone tissue.^76^ Overall, while bulk and pseudobulk expression were highly correlated, differences may reflect cell-type-specific loss in single-cell experiments.

## Discussion

The ScPCA Portal is a downloadable collection of uniformly processed, summarized sc/snRNA-seq data and de-identified metadata from pediatric tumor samples. It is, to our knowledge, the most comprehensive collection of publicly available pediatric tumor single-cell transcriptomic datasets. Users can browse and search the Portal via an intuitive web interface and explore visualizations of individual samples using the UCSC Cell Browser interface.^37^ Summarized data are available at three different processing stages (unfiltered, filtered, or processed objects), allowing users to start from a processed object or perform their own processing. Processed objects contain normalized gene expression data, dimensionality reduction results from PCA and UMAP, cell-type annotations, and CNV inference results. Standardized metadata, containing human-readable values for all fields and ontology term identifiers for a subset of fields, is also provided for all samples. Every library includes a QC report. These processed results and metadata allow researchers to save time by moving directly to downstream analyses, such as identifying marker genes or exploring genes of interest.

Data on the Portal are available as either SingleCellExperiment or AnnData objects for ease of use in the R or Python environment (i.e., Bioconductor or scverse, respectively). The AnnData objects also allow users to integrate ScPCA data with data and tools on other platforms. In particular, our AnnData objects are designed to be mostly compliant with the requirements of CZI CELLxGENE,^5^^,^^6^^,^^77^ and these objects can also be used with Kana.^78^^,^^79^ Data can be downloaded both via the Portal web interface and programmatically using the ScPCAr R package, which is a wrapper for the ScPCA Portal API (https://api.scpca.alexslemonade.org/docs/).

Cell-type annotations are also available from three automated methods: SingleR, CellAssign, and SCimilarity. The first two approaches use publicly available references, while the third uses a foundation model to label cells. These labels are used to construct ontology-aware consensus assignments, which provide harmonized labels across samples.

Many samples on the Portal have additional sequencing data that can also be downloaded, including ADT data from CITE-seq, cell hashing data, bulk RNA-seq, or spatial transcriptomics. These modalities can facilitate expanded analyses. For example, ADT data can help label unknown cell types and correlate RNA to protein expression,^38^ and spatial transcriptomics data can be leveraged to gain more insights about the distribution of cell types in the tissue.^80^ Similarly, users can gain more insight from bulk RNA-seq data available on the Portal by integrating with sc/snRNA-seq data from the same sample.^81^^,^^82^ The sc/snRNA-seq data available on the Portal can also be used to deconvolute existing bulk RNA-seq datasets. The ScPCA Portal therefore enables multimodal comparisons that reveal biological or technical signals that would otherwise not be apparent from a single sequencing modality alone.

We also introduced our open-source and efficient workflow for uniformly processing datasets available on the Portal, scpca-nf. scpca-nf can process raw data from various sequencing types, turning FASTQ files into processed SingleCellExperiment or AnnData objects ready for downstream analyses. Using Nextflow as the framework for scpca-nf with container images for each process makes the workflow modular and portable. Processed output from running scpca-nf on samples from pediatric tumors, cell lines, or other model organisms is eligible for submission to the ScPCA Portal, enabling us to continue to grow the Portal.

Portal data can also support reanalysis of existing pediatric cancer datasets with bulk RNA-seq, such as the Pediatric Brain Tumor Atlas,^83^^,^^84^ allowing researchers to glean more insight from published data without obtaining additional samples. Ultimately, the ScPCA Portal will save researchers time and money, advancing pediatric cancer research.

### Limitations of the study

We note several limitations of the current ScPCA Portal and scpca-nf workflow. First, labeling tumor cells is challenging because neither SingleR nor CellAssign references include them. We mitigate this in several ways. As references are biased toward normal cells, cells without a consensus cell-type annotation are likely to be malignant. We provide CNV estimates for roughly half of Portal libraries, and together with consensus cell types, these can help identify tumor cells, particularly in diagnoses with common copy-number alterations such as neuroblastoma (Figures 5B–5D). We are also augmenting projects through the OpenScPCA project,^68^ which provides robust, transparent annotations that formally distinguish normal from tumor cells and will continue to expand.

Second, although we provide merged objects for each project, we do not provide integrated or batch-corrected objects, as the appropriate correction approach depends on the scientific question and is best left to the user.

Third, while the Portal features several modalities beyond sc/snRNA-seq, not all modalities are currently represented (e.g., single-cell assay for transposase-accessible chromatin using sequencing [scATAC-seq]). Our modular Nextflow workflow is flexibly designed to accommodate additional modalities as the Portal expands.

## Resource availability

### Lead contact

Requests for resources and resource sharing should be directed to the lead contact, Jaclyn N. Taroni (jaclyn.taroni@ccdatalab.org).

### Materials availability

This study did not generate any new material resources.

### Data and code availability

All processed RNA-seq data and de-identified metadata described in this study are freely available through the ScPCA Portal at scpca.alexslemonade.org, which is designed for long-term open access of sc/snRNA-seq and spatial transcriptomics data. Each project, sample, and library is assigned a stable, unique accession number. Raw data (e.g., FASTQ files) are not available for download from the Portal due to the human origins of most samples, which may be subject to controlled-access restrictions. When raw or processed data are also deposited in external sources such as the dbGaP or GEO, the project accession numbers are available from the Portal.

All projects included in this publication are available from the ScPCA Portal with the following accession numbers: SCPCP000001, SCPCP000002, SCPCP000003, SCPCP000004, SCPCP000005, SCPCP000006, SCPCP000007, SCPCP000008, SCPCP000009, SCPCP000010, SCPCP000011, SCPCP000012, SCPCP000013, SCPCP000014, SCPCP000015, SCPCP000016, SCPCP000017, SCPCP000018, SCPCP000020, SCPCP000021, SCPCP000022, SCPCP000023, and SCPCP000024.

Code related to this manuscript is available as follows:

• The Nextflow workflow is available on GitHub at https://github.com/AlexsLemonade/scpca-nf and is archived at Zenodo: https://doi.org/10.5281/zenodo.20072636.^85^
• The ScPCA Portal code can be found at https://github.com/AlexsLemonade/scpca-portal and is archived at Zenodo: https://doi.org/10.5281/zenodo.20058961.^86^
• The benchmarking of tools used to build scpca-nf is available at https://github.com/AlexsLemonade/alsf-scpca/tree/main/analysis and https://github.com/AlexsLemonade/sc-data-integration/tree/main/celltype_annotation, with all repository contents archived at Zenodo: https://doi.org/10.5281/zenodo.20044281^87^ and Zenodo: https://doi.org/10.5281/zenodo.20044314,^88^ respectively.
• All code for creating reference files for consensus cell-type assignment is available at https://github.com/AlexsLemonade/OpenScPCA-analysis/tree/main/analyses/cell-type-consensus, and all repository contents are archived at Zenodo: https://doi.org/10.5281/zenodo.18459136.^89^
• All code to assign OpenScPCA project cell-type annotations is available at https://github.com/AlexsLemonade/OpenScPCA-nf and is archived at Zenodo: https://doi.org/10.5281/zenodo.20056054.^90^
• All code for the ScPCAr package for programmatically downloading data from the Portal can be found at https://github.com/AlexsLemonade/ScPCAr and is archived at Zenodo: https://doi.org/10.5281/zenodo.20044462.^91^
• All code for the underlying figures and analyses is available at https://github.com/AlexsLemonade/scpca-paper-figures and is archived at Zenodo: https://doi.org/10.5281/zenodo.20123383.^92^ To reproduce the figures in this manuscript, see https://github.com/AlexsLemonade/scpca-paper-figures/tree/main/reproduce-figures.

ScPCA documentation describing the downloads available from the Portal is available at https://scpca.readthedocs.io/en/stable/. Any additional information required to reanalyze the data in this study is available from the lead contact upon request.

## Acknowledgments

We thank the data generators and submitters of ScPCA. We also thank Anna Greene for her role in constructing the ScPCA funding opportunity. This work was funded through the Alex’s Lemonade Stand Foundation Childhood Cancer Data Lab and Childhood Cancer Data Lab Postdoctoral Fellowship (S.M.F.).

## Author contributions

Methodology, A.G.H., J.A.S., S.J.S., D.S.M., D.V.P., N.I., A.Y., A.M.G., K.G.W., and J.N.T.; software, A.G.H., J.A.S., S.J.S., D.S.M., D.V.P., N.I., A.Y., A.M.G., K.G.W., and C.J.B.; investigation, A.G.H., J.A.S., S.J.S., and J.N.T.; validation, A.G.H., J.A.S., S.J.S., D.S.M., D.V.P., A.Y., A.M.G., K.G.W., C.J.B., and J.N.T.; formal analysis, A.G.H., J.A.S., and S.J.S.; data curation, A.G.H., J.A.S., S.J.S., D.S.M., A.Y., A.M.G., K.G.W., J.O., and J.N.T.; resources, J.A.S., D.S.M., A.Y., A.M.G., and K.G.W.; writing – original draft, A.G.H., J.A.S., S.J.S., and J.N.T.; writing – review & editing, A.G.H., J.A.S., S.J.S., D.S.M., D.V.P., N.I., A.Y., A.M.G., K.G.W., C.J.B., S.M.F., J.O., C.S.G., and J.N.T.; visualization, A.G.H., J.A.S., S.J.S., D.V.P., and J.N.T.; supervision, J.O., C.S.G., and J.N.T.; conceptualization, C.S.G. and J.N.T.; project administration, C.S.G. and J.N.T.

## Declaration of interests

A.G.H., J.A.S., S.J.S., D.S.M., D.V.P., N.I., A.Y., A.M.G., K.G.W., C.J.B., J.O., and J.N.T. are or were employees of Alex’s Lemonade Stand Foundation, a sponsor of this research.

## STAR★Methods

### Key resources table

**Table:** 

REAGENT or RESOURCE	SOURCE	IDENTIFIER
**Deposited data**		

Summarized gene expression data	This paper	https://scpca.alexslemonade.org

**Software and algorithms**		

scpca-nf workflow used for processing all ScPCA Portal data	This paper	https://github.com/AlexsLemonade/scpca-nf; Zenodo: https://doi.org/10.5281/zenodo.20072636^85^
ScPCA Portal code	This paper	https://github.com/AlexsLemonade/scpca-portal; Zenodo: https://doi.org/10.5281/zenodo.20058961^86^
Benchmarking of tools used to build scpca-nf	This paper	https://github.com/AlexsLemonade/alsf-scpca (Zenodo: https://doi.org/10.5281/zenodo.20044281)^87^ and https://github.com/AlexsLemonade/sc-data-integration (Zenodo: https://doi.org/10.5281/zenodo.20044314) ^88^
Code for creating reference files for consensus cell type assignment	This paper	https://github.com/AlexsLemonade/OpenScPCA-analysis; Zenodo: https://doi.org/10.5281/zenodo.18459136^89^
Workflow for assigning OpenScPCA project cell type annotations to ScPCA data	This paper	https://github.com/AlexsLemonade/OpenScPCA-nf; Zenodo: https://doi.org/10.5281/zenodo.20056054^90^
ScPCAr package for programmatically downloading from the Portal	This paper	https://github.com/AlexsLemonade/ScPCAr; Zenodo: https://doi.org/10.5281/zenodo.20044462^91^
Code for underlying figures and analyses	This paper	https://github.com/AlexsLemonade/scpca-paper-figures; Zenodo: https://doi.org/10.5281/zenodo.20123383^92^
Nextflow	Tomasso et al.^14^	https://github.com/nextflow-io/nextflow/tree/master
salmon	Patro et al.^59^	https://anaconda.org/channels/bioconda/packages/salmon/overview
alevin-fry	He et al.^15^	https://anaconda.org/channels/bioconda/packages/alevin-fry/overview
Space Ranger	10x Genomics	https://www.10xgenomics.com/support/software/space-ranger/latest
SingleCellExperiment	Amezquita et al.^12^	https://bioconductor.org/packages/release/bioc/html/SingleCellExperiment.html
anndata	Virshup et al.^56^	https://pypi.org/project/scvi-tools/
DropletUtils	Lun et al.^47^; Griffiths et al.^48^	https://bioconductor.org/packages/release/bioc/html/DropletUtils.html
miQC	Hippen et al.^49^	https://bioconductor.org/packages/release/bioc/html/miQC.html
scDblFinder	Germain et al.^93^	https://bioconductor.org/packages/release/bioc/html/scDblFinder.html
scuttle	McCarthy et al.^94^	https://bioconductor.org/packages/release/bioc/html/scuttle.html
scran	Lun et al.^95^	https://bioconductor.org/packages/release/bioc/html/scran.html
scater	McCarthy et al.^94^	https://bioconductor.org/packages/release/bioc/html/scater.html
Seurat	Hao et al.^96^	https://satijalab.org/seurat/
vireo	Huang et al.^97^; Weber et al.^57^	https://github.com/single-cell-genetics/vireo
batchelor	Lun et al.^98^	https://bioconductor.org/packages/release/bioc/html/batchelor.html
SingleR	Aran et al.^52^	https://bioconductor.org/packages/release/bioc/html/SingleR.html
celldex	Aran et al.^52^	https://bioconductor.org/packages/release/data/experiment/html/celldex.html
CellAssign	Zhang et al.^53^	https://docs.scvi-tools.org/en/stable/installation.html
SCimilarity	Heimberg et al.^54^	https://genentech.github.io/scimilarity/index.html
inferCNV	inferCNV of the Trinity CTAT Project^55^	https://www.bioconductor.org/packages/release/bioc/html/infercnv.html
zellkonverter	Zappia et al.^99^	https://bioconductor.org/packages/release/bioc/html/zellkonverter.html
DESeq2	Love et al.^100^	https://bioconductor.org/packages/release/bioc/html/DESeq2.html
lme4	Bates et al.^101^	https://cran.r-project.org/web/packages/lme4/index.html
ontoProc	Carey^102^	https://bioconductor.org/packages/release/bioc/html/ontoProc.html

**Other**		

HsapDv	Ontology Lookup Service^16^	https://www.ebi.ac.uk/ols4/ontologies/hsapdv
PATO	Gkoutos et al.^17^	https://www.ebi.ac.uk/ols4/ontologies/pato
NCBI Taxonomy	Schoch et al.^19^	https://www.ncbi.nlm.nih.gov/taxonomy
Mondo	Vasilevsky et al.^21^	https://www.ebi.ac.uk/ols4/ontologies/mondo
UBERON	Haendel et al.^23^	https://www.ebi.ac.uk/ols4/ontologies/uberon
Hancestro	Morales et al.^26^	https://www.ebi.ac.uk/ols4/ontologies/hancestro
Cell Ontology	Bard et al.^64^; Meehan et al.^63^; Diehl et al.^62^; Tan et al.^103^	https://www.ebi.ac.uk/ols4/ontologies/cl
Blueprint and ENCODE	Martens et al.^104^; The ENCODE Consortium^105^	https://rdrr.io/github/LTLA/celldex/man/BlueprintEncodeData.html
PanglaoDB	Franzén et al.^61^	https://panglaodb.se/
CellMarker 2.0	Hu et al.^65^	http://117.50.127.228/CellMarker/

### Experimental model and study participant details

Data on the ScPCA Portal were generated and compiled by each contributing lab and institution. As of May 1, 2026, gene expression data from 704 samples are available. This includes 581 human patient samples, 117 samples from patient-derived xenografts, 4 samples from immortalized human cell lines, and 2 samples from cell lines passaged from patient-derived xenografts representing 55 different pediatric cancer types.

#### Metadata

Submitters were required to submit the age, sex, organism, diagnosis, subdiagnosis (if applicable), disease timing (e.g., initial diagnosis) and tissue of origin for each sample. The submitted metadata was standardized across projects, including converting all ages to years, removing abbreviations used in diagnosis, subdiagnosis, or tissue of origin, and using standard values across projects as much as possible for diagnosis, subdiagnosis, disease timing, and tissue of origin. For example, all samples obtained at diagnosis were assigned the value Initial diagnosis for disease timing.

In an effort to ensure sample metadata for ScPCA are compatible with CZI’s CELLxGENE, ontology term identifiers were assigned to metadata categories for each sample following the guidelines present in the CELLxGENE schema,^106^^,^^107^ as shown in Table 1.

The majority (87%) of projects on the Portal have additional metadata fields, such as the presence or absence of treatment, tumor grade, or whether a sample was obtained from a primary tumor or metastasis.

#### Ethics statement

For ALSF-funded datasets comprised of human subjects data, Institutional Review Boards (IRB) or research ethics boards at grantee institutions approved the research or determined it was exempt. For community-contributed datasets containing summarized data and de-identified metadata from human subjects, submitting institutions certified that all approvals and consents were obtained or listed the IRB protocol in transfer agreements. ALSF-funded xenograft datasets were approved by the grantee institution’s Institutional Animal Care and Use Committee.

### Method details

#### Data generation and processing

Raw data and metadata were generated and compiled by each lab and institution contributing to the Portal. Single-cell or single-nuclei libraries were generated using one of the commercially available kits from 10x Genomics. For bulk RNA-seq, RNA was collected and sequenced using either paired-end or single-end sequencing. For spatial transcriptomics, cDNA libraries were generated using the Visium kit from 10x Genomics. All libraries were processed using our open-source pipeline, scpca-nf, to produce summarized gene expression data. A detailed summary with the total number of samples and libraries collected for each sequencing method broken down by project is available in Table S1.

#### Processing single-cell and single-nuclei RNA-seq data with alevin-fry

To quantify RNA-seq gene expression for each cell or nucleus in a library, scpca-nf uses salmon alevin^108^ and alevin-fry^15^ to generate a gene-by-cell counts matrix. Prior to mapping, we generated an index using transcripts from both spliced cDNA and unspliced cDNA sequences, denoted as the splici index.^15^ The index was generated from the human genome, GRCh38, Ensembl version 104. salmon alevin was run using selective alignment to the splici index with the --rad option to generate a reduced alignment data (RAD) file required for input to alevin-fry.

The RAD file was used as input to the recommended alevin-fry workflow, with the following customizations. At the generate-permit-list step, we used the --unfiltered-pl option to provide a list of expected barcodes specific to the 10x kit used to generate each library. The quant step was run using the cr-like-em resolution strategy for feature quantification and UMI de-duplication.

#### Post alevin-fry processing of single-cell and single-nuclei RNA-seq data

The output from running alevin-fry includes a gene-by-cell counts matrix, with reads from both spliced and unspliced reads for all potential cell barcodes. The gene-by-cell counts matrix is read into R to create a SingleCellExperiment object using fishpond::load_fry(). The resulting object contains a counts assay with a gene-by-cell counts matrix where all spliced and unspliced reads for a given gene are totaled together. We also include a spliced assay that contains a gene-by-cell counts matrix with only spliced reads. These matrices include all potential cells, including empty droplets, and are provided for all Portal downloads in the unfiltered objects saved as.rds files with the _unfiltered.rds suffix.

Each droplet was tested for deviation from the ambient RNA profile using DropletUtils::emptyDropsCellRanger()^47^^,^^48^ and those with an FDR ≤0.01 were retained as likely cells. If a library did not have a sufficient number of droplets and DropletUtils::emptyDropsCellRanger() failed, cells with fewer than 100 UMIs were removed. Gene expression data for any cells that remain after filtering are provided in the filtered objects saved as.rds files with the _filtered.rds suffix. These filtered objects additionally contain results from doublet detection performed with scDblFinder::scDblFinder(),^93^ including each cell’s predicted class (“singlet” or “doublet”) as well as the associated doublet score. However, predicted doublets were not filtered out; users can instead use these scDblFinder results to filter doublets as needed for their specific analysis needs.

Following removal of empty droplets, scpca-nf proceeds to remove cells that are likely to be compromised by damage or low-quality sequencing. miQC was used to calculate the posterior probability that each cell is compromised.^49^ Any cells with a probability of being compromised greater than 0.75 and fewer than 200 genes detected were removed before further processing. The gene expression counts from the remaining cells were log-normalized using the deconvolution method from Lun, Bach, and Marioni.^50^ Briefly, scran::quickCluster() was used to derive cell clusters on which to calculate sum factors with scran::computeSumFactors(), which are in turn used during normalization with scuttle::logNormCounts(). If this deconvolution-based approach failed for any reason, only scuttle::logNormCounts() was used for normalization.

Next, scran::modelGeneVar() was used to model gene variance from the log-normalized counts and scran::getTopHVGs() was used to select the top 2000 high-variance genes. These were used as input to calculate the top 50 principal components using scater::runPCA(). Finally, UMAP embeddings were calculated from the principal components with scater::runUMAP(). The raw and log-normalized counts, list of 2000 high-variance genes, principal components, and UMAP embeddings are all stored in the processed objects saved as.rds files with the _processed.rds suffix.

#### Quantifying gene expression for libraries with CITE-seq or cell hashing

All libraries with antibody-derived tags (ADTs) or hashtag oligonucleotides (HTOs) were mapped to a reference index using salmon alevin and quantified using alevin-fry. The reference indices were constructed using the salmon index command with the --feature option. References were custom-built for each ScPCA project and constructed using the submitter-provided list of ADTs or HTOs and their barcode sequences.

The ADT-by-cell or HTO-by-cell counts matrix produced by alevin-fry were read into R as a SingleCellExperiment object and saved as an alternative experiment (altExp) in the same SingleCellExperiment object with the unfiltered gene expression counts data. The altExp within the unfiltered object contains all identified ADTs or HTOs and all barcodes identified in the RNA-seq gene expression data. Any barcodes that only appeared in either ADT or HTO data were discarded, and cell barcodes that were only found in the gene expression data (i.e., did not appear in the ADT or HTO data) were assigned zero counts for all ADTs and HTOs. Any cells removed after filtering empty droplets were also removed from the ADT and HTO counts matrices and before creating the filtered SingleCellExperiment object.

#### Processing ADT expression data from CITE-seq

The ADT count matrix stored in the unfiltered object was used to calculate an ambient profile with DropletUtils::ambientProfileEmpty(). The ambient profile was used to calculate quality-control statistics with DropletUtils::cleanTagCounts() for all cells remaining after removing empty droplets. Any negative or isotype controls were taken into account when calculating QC statistics. This function flags cells as low-quality if they either have very high levels of ambient contamination and/or negative/isotype controls (if present), or lack ambient expression altogether, which may indicate failed capture. However, we did not remove any cells based on ADT quality because that would remove those cells from the SingleCellExperiment object, regardless of the quality of the RNA expression. Instead, the filtered and processed objects contain the results from running DropletUtils::cleanTagCounts(), which users can leverage for filtering as desired.

ADT count data were then normalized using scuttle::computeMedianFactors(), which calculates a per-cell size factor as the median ratio of the cell’s counts to the background profile previously calculated with DropletUtils::ambientProfileEmpty(). We then used these factors to normalize ADT counts with scuttle::logNormCounts(). If median-based normalization failed for any reason, ADT counts were log-transformed after adding a pseudocount of 1. We only performed normalization on cells that would be retained after ADT filtering; we assigned NA normalized counts to any cells that would be filtered out based on DropletUtils::cleanTagCounts(). The normalized ADT data are available in the altExp of the processed object. Although scpca-nf normalizes ADT counts, the workflow does not perform any dimensionality reduction of ADT data; only the RNA counts data are used as input for dimensionality reduction. Additionally, note that we did not perform background subtraction on the ADT counts, but we provide the ambient profile calculated with DropletUtils::ambientProfileEmpty(), which users can employ to perform global de-noising as needed. During conversion to AnnData objects, the modalities are exported as separate RNA (_rna.h5ad) and ADT (_adt.h5ad) objects.

#### Processing HTO data from multiplexed libraries

As with ADT data, scpca-nf does not filter any cells based on HTO expression, and any cells removed after filtering empty droplets based on the unfiltered RNA counts matrix are also removed from the HTO counts matrix in the filtered object. scpca-nf does not perform any additional filtering or processing of the HTO-by-cell counts matrix, so the same filtered matrix is included in the processed object.

To identify which cells come from which sample in a multiplexed library, we applied three different demultiplexing methods: genetic demultiplexing, HTO demultiplexing using DropletUtils::hashedDrops(), and HTO demultiplexing using Seurat::HTODemux(). We do not provide separate SingleCellExperiment objects for each sample in a library. Each multiplexed library object contains the counts data from all samples and the results from all three demultiplexing methods to allow users to select which method(s) to use.

#### Genetic demultiplexing

If all samples in a multiplexed library were also sequenced using bulk RNA-seq, we performed genetic demultiplexing using genotype data from both bulk RNA-seq and single-cell or single-nuclei RNA-seq.^57^ If bulk RNA-seq was not available, no genetic demultiplexing was performed.

Bulk RNA-seq reads for each sample were mapped to a reference genome using STAR,^109^ and multiplexed single-cell or single-nuclei RNA-seq reads were mapped to the same reference genome using STARsolo.^110^ The mapped bulk reads were used to call variants and assign genotypes with bcftools mpileup.^111^ cellsnp-lite was then used to genotype single-cell data at the identified sites found in the bulk RNA-seq data.^112^ Finally, vireo was used to identify the sample of origin.^97^

#### HTO demultiplexing

For all multiplexed libraries, we performed demultiplexing using DropletUtils::hashedDrops() and Seurat::HTODemux(). For both methods, we used the default parameters and only performed demultiplexing on the filtered cells present in the filtered object. The results from both these methods are available in the filtered and processed objects.

#### Quantification of spatial transcriptomics data

10x Genomics’ Space Ranger^60^ was used to quantify gene expression data from spatial transcriptomics libraries. cellranger mkref was used to create a reference index from the human genome, GRCh38, Ensembl version 104. The FASTQ files, microscopic slide image, and slide serial number were provided as input to spaceranger count. The raw and filtered counts matrix, slide images, and the summary report output by spaceranger count are included in the output from scpca-nf.

Quantification of bulk RNA-seq data

fastp was used to trim adapters and perform quality and length filtering on all FASTQ files from bulk RNA-seq. We used a decoy-aware reference created from spliced cDNA sequences with the entire human genome sequence (GRCh38, Ensembl version 104) as the decoy.^59^ The trimmed reads were then provided as input to salmon quant for selective alignment. In addition to using the default parameters for salmon quant, we applied the --seqBias and --gcBias flags to correct for sequence-specific biases due to random hexamer priming and fragment-level GC biases, respectively.

#### Cell type annotation

Cell type labels determined by SingleR,^52^ CellAssign,^53^ and SCimilarity^54^ were added to processed SingleCellExperiment objects. If cell types were obtained from the submitter of the dataset, the submitter-provided annotations were incorporated into all SingleCellExperiment objects (unfiltered, filtered, and processed).

To prepare the references used for assigning cell types, we developed a separate workflow, build-celltype-index.nf, within scpca-nf. We used the BlueprintEncodeData reference from the celldex package^52^^,^^104^^,^^105^ to train the SingleR classification model with SingleR::trainSingleR(). In the main scpca-nf workflow, this model and the processed SingleCellExperiment object were input to SingleR::classifySingleR(). The SingleR output of cell type annotations and a score matrix for each cell and all possible cell types were added to the processed SingleCellExperiment object.

The BlueprintEncodeData reference was chosen because it had either a similar or higher delta median statistic compared to other references available in celldex when applied to samples from a variety of diagnoses. The delta median statistic for each cell was calculated by subtracting the median cell type score from the score associated with the assigned cell type^113^ and was used to evaluate confidence in SingleR cell type assignments. The cell type report shows the distribution of delta median values for each cell type. A higher delta median statistic for a cell generally indicates higher confidence in the final cell type annotation.

For CellAssign, marker gene references were created using the marker gene lists available on PanglaoDB.^61^ Organ-specific references were built using all cell types in a specified organ listed in PanglaoDB to accommodate all ScPCA projects encompassing a variety of disease and tissue types. If a set of disease types in a given project encompassed cells that may be present in multiple organ groups, multiple organs were combined. Since many cancers may have infiltrating immune cells, all immune cells were also included in each organ-specific reference. For example, we created a reference containing bone, connective tissue, smooth muscle, and immune cells for sarcomas that appear in bone or soft tissue. The specific reference information and list of organs included in that reference for a given library is available in the metadata of each processed object.

Given the processed SingleCellExperiment object and organ-specific reference, scvi.external.CellAssign() was used in the main scpca-nf workflow to train the model and predict the assigned cell type. For each cell, CellAssign calculates a probability of assignment to each cell type in the reference. The probability matrix and a prediction based on the most probable cell type were added as cell type annotations to the processed SingleCellExperiment object. We also display the distribution of all probabilities calculated by CellAssign in the cell type report; more confident labels are expected to have many values close to 1.

For SCimilarity, the foundation model described in Heimberg et al.^54^ containing 7.3 million cells from various normal and diseased tissues was obtained from Zenodo^114^ and used to annotate cells in all samples. Embeddings were first computed on the processed AnnData objects using scimilarity.get_embeddings() followed by cell type prediction using scimilarity.get_predictions_knn() with weighting=True. The assigned cell type label and the distance of the query cell to the closest cell in the model were added to the processed SingleCellExperiment object. A plot showing the distribution of the distance metric calculated by SCimilarity is present in the cell type report. Distances larger than 0.05 can indicate that the model is less confident in the prediction.

#### Assigning consensus cell types

Cell type labels obtained from SingleR, CellAssign, and SCimilarity were then used to assign an ontology-aware consensus cell type label. We first assigned each of the cell types present in the PanglaoDB^61^ reference used with CellAssign to an appropriate Cell Ontology term.^62^^,^^63^^,^^64^^,^^103^ For cell types available in the BlueprintEncodeData reference used with SingleR and the foundation model used with SCimilarity, we used the provided Cell Ontology terms.

We then created a reference table containing all possible combinations of cell types assigned using SingleR, CellAssign, and SCimilarity. Consensus cell types are assigned if two of the three annotations share a latest common ancestor (LCA), identified using ontoProc::findCommonAncestors(),^102^ that meets the following criteria. Otherwise, no consensus cell type is assigned, and the cell is labeled as “Unknown”.

1. The terms share at least 1 LCA that either has fewer than 170 descendants or is one of “neuron”, “epithelial cell”, “columnar/cuboidal epithelial cell”, or “endo-epithelial cell”.
2. If more than 1 LCA is shared between two terms, then the LCA with the fewest descendants is kept and all others are discarded.
3. If the LCA has fewer than 170 descendants and is one of the following non-specific LCA terms, no consensus cell type is assigned: “bone cell”, “lining cell”, “blood cell”, “progenitor cell”, “supporting cell”, “biogenic amine secreting cell”, “protein secreting cell”, “extracellular matrix secreting cell”, “serotonin secreting cell”, “peptide hormone secreting cell”, “exocrine cell”, “sensory receptor cell”, or “interstitial cell”.

If more than one LCA is identified as a possible consensus cell type, meaning there is agreement among all three methods, the LCA with the fewest descendants is used as the consensus cell type.

The consensus cell type assignments, including both the Cell Ontology term and the associated human-readable name, are available in processed object files on the Portal.

Consensus cell type assignments were evaluated by looking at marker gene expression in a set of cell-type specific marker genes. Marker genes were obtained from the list of Human cell markers on CellMarker 2.0.^65^ We considered only those that are specific to a single cell type, with the exception of hematopoietic precursor cells, which express genes found in other, more differentiated immune cells.

#### Cell types annotated as part of the OpenScPCA project

As part of the ongoing OpenScPCA project,^68^ cell types for each project are manually annotated to label disease-specific cell types or cell states. After annotations for all samples in a given project have been validated, they are added to all SingleCellExperiment objects (unfiltered, filtered, and processed) for that project on the Portal. To date, cell types have been assigned and validated for SCPCP000004 (neuroblastoma) and SCPCP000015 (Ewing sarcoma). The approaches for cell type annotation were originally developed in the OpenScPCA-analysis GitHub repository^115^ in the cell-type-neuroblastoma-04 and cell-type-ewings analysis modules, respectively. These analysis modules provide full information on the specific approaches used for annotation. The cell type annotations included in the ScPCA Portal were subsequently generated in corresponding Nextflow modules in the OpenScPCA-nf GitHub repository.^116^

For SCPCP000004 (neuroblastoma), shown in Figure 5, cell type annotation is performed with both SingleR^69^ and scANVI/scArches^117^ using the NBAtlas from Bonine et al. as a ref. 69. Final annotations are derived based on agreement between these two methods and the consensus cell types. If SingleR and scANVI/scArches agree exactly, then that label is used. If SingleR and scANVI/scArches labels are in same broad family (e.g., T cell and CD4 + T cell) then the broad family label is used (e.g., T cell). If SingleR and scANVI/scArches disagree and at least one inference agrees with the consensus cell type label then we assign that label. If the consensus cell type is Unknown and one of the SingleR and/or scANVI/scArches labels is one of Neuroendocrine and the other is one of Schwann, Stromal other, or Fibroblast, the Neuroendocrine label is assigned.

For SCPCP000015 (Ewing sarcoma), tumor cells were first identified by running AUCell^118^ on a merged object containing all samples with EWS::FLI1 marker gene sets obtained from MSigDB and published literature. Cells with an AUC >0.4 for the EWS::FLI1 marker gene set obtained from Aynaud et al.^119^ and AUC >0.1 for the STAEGE_EWING_FAMILY_TUMOR gene set from MSigDB^120^ were classified as tumor EWS-high. Cells with an AUC >0.1 for the EWS::FLI1 marker gene set obtained from Wrenn et al.^121^ and AUC >0.05 for the HALLMARK_EPITHELIAL_MESENCHYMAL_TRANSITION gene set from MSigDB^122^ were classified as tumor EWS-low. Cells that met the criteria for tumor EWS-high and had mean expression of proliferative markers (MKI67, PCNA, and TOP2A) > 0 were classified as tumor EWS-high proliferative. For all non-tumor cells, the consensus cell type label was used.

#### Copy-number variation inference

We used inferCNV^55^ with the i6 HMM to estimate copy-number variation (CNV) events for each library, for each chromosome arm. We designated a set of normal consensus cell types to use for each library’s normal reference based on the given sample’s diagnosis. The list of cell types included in the reference used for inferCNV can be found in the metadata of the processed object for a given library. All libraries were processed with inferCNV except: i) libraries without assigned consensus cell types, ii) libraries with fewer than 100 normal reference cells, and iii) libraries from non-cancerous samples. We calculated the total CNVs per cell using the feature output from the i6 HMM by summing CNV calls across all chromosome arms.

#### Generating merged data

Merged objects are created with the merge.nf workflow within scpca-nf. This workflow takes as input the processed SingleCellExperiment objects in a given ScPCA project output by scpca-nf and creates a single merged SingleCellExperiment object containing gene expression data and metadata from all libraries in that project. The merged object includes both raw and normalized counts for all cells from all libraries. Because the same reference index was used to quantify all single-cell and single-nuclei RNA-seq data, the set of genes is the same in the merged object and the individual objects. Library-, cell- and gene-specific metadata from each of the processed SingleCellExperiment objects are also combined and stored in the merged object. The merge.nf workflow does not perform batch correction or integration, so the counts in the merged object are not batch-corrected.

The top 2000 shared high-variance genes are identified from the merged counts matrix by modeling variance using scran::modelGeneVar() and specifying library IDs for the block argument. These genes are used to calculate library-aware principal components with batchelor::multiBatchPCA().^98^ The top 50 principal components were selected and used to calculate UMAP embeddings for the merged object.

If any libraries included in the ScPCA project contain additional ADT data, the raw and normalized ADT data are also merged and stored in the altExp slot of the merged SingleCellExperiment object. If the merged object contains an altExp with merged ADT data, two AnnData objects are exported to create separate RNA (_rna.h5ad) and ADT (_adt.h5ad) objects.

If any libraries in the ScPCA project are multiplexed and contain HTO data, no merged object is created due to potential ambiguity in identifying samples across multiplexed libraries. Merged objects were not created for projects with more than 100 samples because of the computational resources required to work with them.

#### Converting SingleCellExperiment objects to AnnData objects

zellkonverter::writeH5AD()^99^ was used to convert SingleCellExperiment objects to AnnData format and export the objects as.h5ad files. For any SingleCellExperiment objects containing an altExp (e.g., ADT data), the RNA and ADT data were exported and saved separately as RNA (_rna.h5ad) and ADT (_adt.h5ad) files. Multiplexed libraries were not converted to AnnData objects, due to the potential for ambiguity in sample origin assignments.

All merged SingleCellExperiment objects were converted to AnnData objects and saved as.h5ad files. If a merged SingleCellExperiment object contained any ADT data, the RNA and ADT data were exported and saved separately as RNA (_rna.h5ad) and ADT (_adt.h5ad) objects. In contrast, if a merged SingleCellExperiment object contained HTO data due to the presence of any multiplexed libraries in the merged object, the HTO data was removed from the SingleCellExperiment object and not included in the exported AnnData object.

### Quantification and statistical analysis

Details regarding total sample and/or cell numbers and any statistical tests used can be found in the figure legends and figures. For Figures 4 and 5; Figures S4 and S5, numbers in parentheses in the figure indicate total cell numbers. For Figures 1 and 6; Figure S7, numbers in parentheses in the figure indicate total sample numbers.

#### Benchmarking of alevin-fry and cellranger count performance

Six libraries, three single-cell and three single-nuclei, were randomly selected and used to benchmark the performance of alevin-fry and cellranger count, results of which are shown in Figure S1. Libraries were processed with salmon alevin v1.5.2 and alevin-fry v0.4.1 or cellranger count from Cell Ranger v6.1.2. Results were generated using default parameters for single-cell libraries and use of the --include_introns flag to include intronic reads for single-nuclei libraries only. The Pearson correlation between mean gene expression across both methods is reported in Figure S1B.

#### Analysis of bulk RNA-seq data

##### Data preparation

We identified solid tumor samples with both bulk and single-cell (or single-nuclei) RNA-seq data in the ScPCA Portal for analysis, with multiplexed samples excluded (*N* = 105). We removed low-quality samples based on visual inspection of quality control reports (*N* = 8), leaving a total of 97 samples across five ScPCA projects for analysis.

For each project, we transformed and normalized bulk counts matrices for all samples using DESeq2::rlog().^100^ We obtained pseudobulk counts by summing raw single-cell counts for each sample, and similarly transformed each project’s resulting counts matrix with DESeq2::rlog(). We filtered out genes which were not observed in either the bulk or pseudobulk raw counts matrices before subsequent analysis. For each project, we then used the lme4 R package^101^ to construct a linear model predicting bulk from pseudobulk counts considering a random effect for sample id: bulk ∼ pseudobulk + (1|sample_id).

##### Overrepresentation analysis

We next conducted overrepresentation analysis (ORA) to ascertain whether certain cell types might be overrepresented either modality (bulk vs. pseudobulk). We specifically tested overrepresentation of the PanglaoDB cell type marker gene sets used for each project’s respective CellAssign reference.

For input to the ORA, we summarized model residuals within each project by taking the median residual for each gene across samples and then transformed these summarized residuals into Z-scores. We identified outlier genes as those with Z-scores greater than 2.5 (positive outliers) or less than −2.5 (negative outliers). In this case, positive outliers represent genes with comparatively higher expression in the bulk modality, and negative outliers represent genes with comparatively higher expression in the single-cell modality.

For each set of cell type marker genes, we calculated two odds ratios representing whether genes were overrepresented in the positive outliers (enriched in bulk) or negative outliers (enriched in pseudobulk). We calculated *p*-values for both the bulk and pseudobulk enrichment directions via permutation testing with 10,000 replicates. We defined gene sets with significant overrepresentation as those with a false-discovery-rate-corrected *p*-value ≤ 0.05.^123^

### Additional resources

Documentation for the ScPCA Portal can be found at https://scpca.readthedocs.io.

## Supplemental information

Document S1. Figures S1–S7

Table S1. Overview of ScPCA Portal datasets, related to Figure 1 This table provides descriptions and sample and library counts for projects in the ScPCA Portal. scpca_project_id: ScPCA project unique identifier. Diagnosis group: diagnosis group as shown in Figure 1A. Diagnoses: full set of diagnoses for all samples in the project. Total number of samples (S): number of samples in the project. Total number of libraries (L): number of libraries in the project. Due to additional sequencing modalities and/or multiplexing, projects may have more libraries than samples. All remaining columns give the number of libraries, as designated with (L), with the given suspension type, 10× kit version, or additional modality.

Table S2. Summary of references used for cell-type annotation with CellAssign, related to Figure 3 This table summarizes the references used for assigning cell types using CellAssign. References were built using all cell types from a set of organs in PanglaoDB’s marker gene list. scpca_project_id: ScPCA project unique identifier. Diagnoses: full set of diagnoses for all samples in the project. ScPCA reference name: custom reference name. PanglaoDB organs included in reference: organs from PanglaoDB included in the reference. Marker genes for all cell types in each organ were included.

Document S2. Transparent peer review records for Hawkins et al

Document S3. Article plus supplemental information
