# Supplementary material for: The Single-Cell Pediatric Cancer Atlas: Data portal and open-source tools for single-cell transcriptomics of pediatric tumors
Source: Cell Genom. 2026 Jun 24;6(7):101283. doi: 10.1016/j.xgen.2026.101283 (PMC13347941; doi:10.1016/j.xgen.2026.101283)
Supplement: Document S1. Figures S1–S7 [file mmc1.pdf]

Cell Genomics, Volume *6*

Supplemental information

The Single-Cell Pediatric Cancer Atlas:

Data portal and open-source tools for single-cell

transcriptomics of pediatric tumors

Allegra G. Hawkins, Joshua A. Shapiro, Stephanie J. Spielman, David S. Mejia, Deepashree Venkatesh Prasad, Nozomi Ichihara, Arkadii Yakovets, Avrohom M. Gottlieb, Kurt G. Wheeler, Chante J. Bethell, Steven M. Foltz, Jennifer O'Malley, Casey S. Greene, and Jaclyn N. Taroni

Supplementary Figures

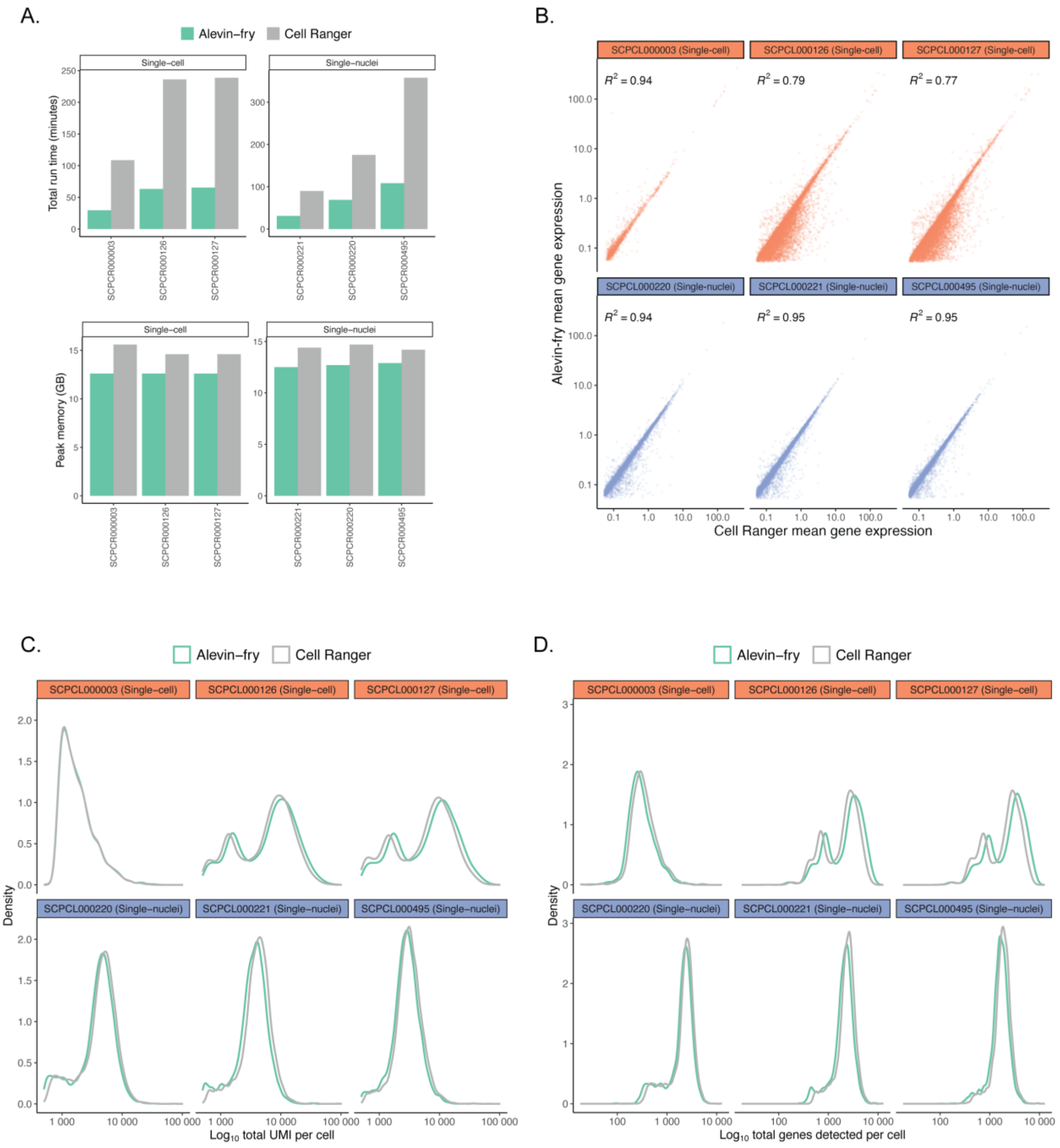

Figure S1: ***Results from benchmarking alevin-fry and cellranger count performance, Related to Figure 2.***

Panels compare metrics for six ScPCA libraries (three single-cell and three single-nuclei), obtained from processing with salmon alevin and alevin-fry [S1] or cellranger count. Results were generated with Cell Ranger v6.1.2 [S2, S3] using default parameters for single-cell libraries and the --include_introns flag to include intronic reads for single-nuclei libraries only. Libraries were processed with salmon alevin v1.5.2

and alevin-fry v0.4.1 using an index containing both spliced and unspliced cDNA (see Methods). Libraries used for benchmarking were randomly chosen.

A. Runtime in minutes (top row) and peak memory in GB (bottom row) for libraries processed with both platforms. Processing with alevin-fry was consistently faster and more memory-efficient than processing with cellranger count.

Panels B-D show only cells present in both alevin-fry and cellranger count outputs.

B. Comparison of mean gene expression values for libraries processed with both platforms, shown on a log-scale. Each point is a gene, and only genes detected in at least 5 cells are shown. R^2 values shown in the top left corner of each panel reflect broad agreement in mean gene expression values between platforms.

C. Comparison of log total UMI counts for libraries processed with both platforms. The total UMI count per cell between platforms broadly agree, although alevin-fry returned slightly higher values for certain single-cell libraries.

D. Comparison of log total genes detected per cell for libraries processed with both platforms. The total number of genes detected per cell between platforms broadly agree, although alevin-fry returned slightly higher values for certain single-cell libraries.

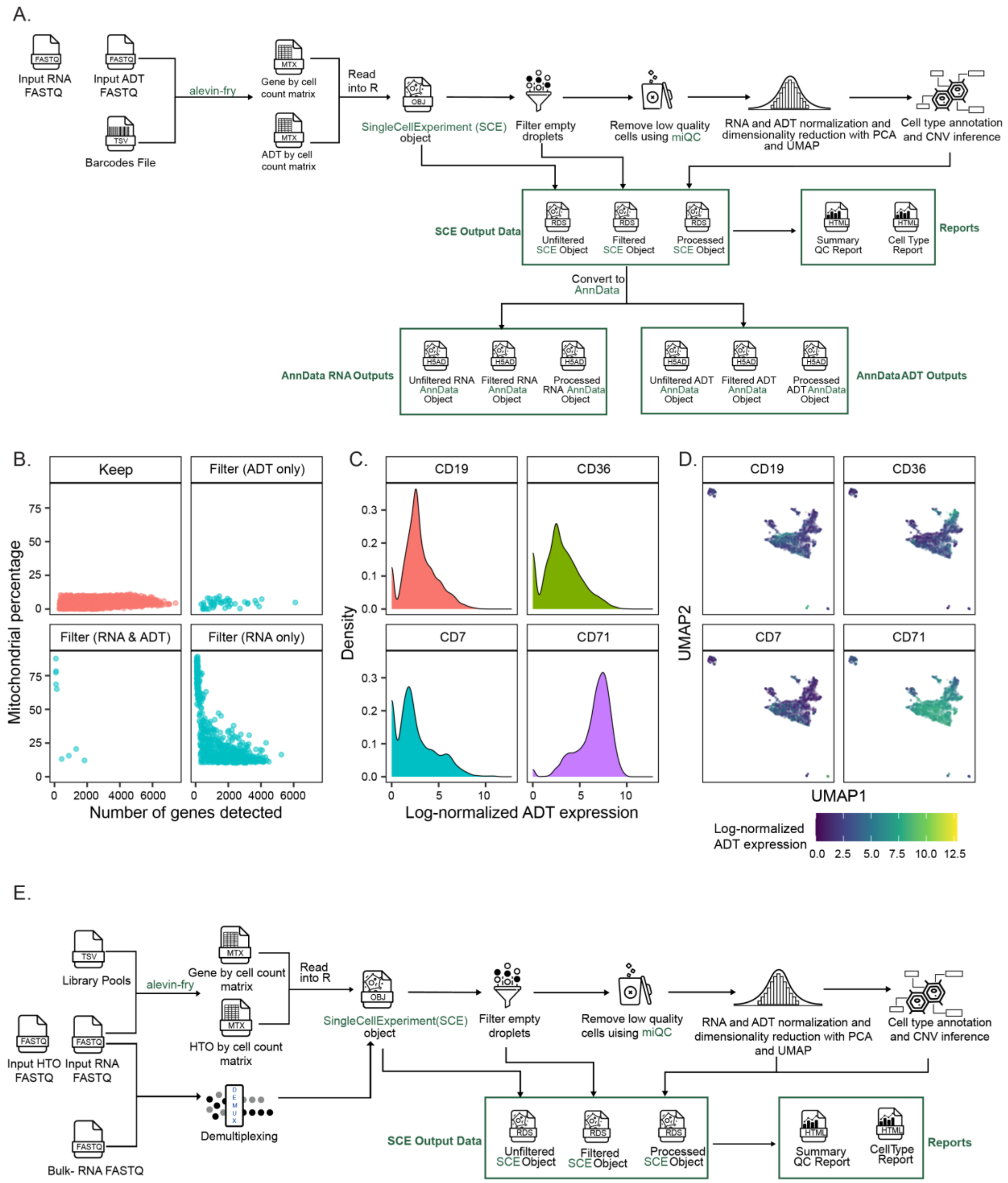

Figure S2: ***Processing additional single-cell modalities in scpca-nf, Related to Figure 2.***

A. Overview of the scpca-nf workflow for processing libraries with CITE-seq or antibody-derived tag (ADT) data. The workflow mirrors that shown in Figure 2A with several differences accounting for the presence of ADT data. First, both an RNA and ADT FASTQ file are required as input to alevin-fry, along with a TSV file containing information about ADT barcodes. Second, during post-processing, statistics are calculated to filter cells based on ADT counts, but the filter is not applied. ADT counts are also normalized and included in the Processed SCE Object. Third, the summary QC report will include a CITE-seq section with additional information about ADT-level processing. Fourth, the workflow exports SCE objects containing both RNA and ADT results, while separate AnnData [S4] objects for RNA and ADT are exported.

Panels B-D show example figures that appear in the CITE-seq section of the summary QC report, shown here for SCPCL000290.

B. The percent of mitochondrial reads in each cell against the number of genes detected in each cell. The panel labeled “Keep” displays cells that are retained based on both RNA and ADT counts. Other panels display cells that are filtered based only on the given type of counts.

C. Density plots of the log-normalized ADT counts for the library’s four most variable ADTs.

D. UMAP log-normalized RNA expression values. Cells are colored by expression of the given highly-variable ADT.

E. Overview of the scpca-nf workflow for multiplexed libraries. The workflow mirrors that shown in Figure 2A with several differences accounting for the presence of multiplexed data. First, both an RNA and HTO FASTQ file are required as input to alevin-fry, along with a TSV file providing information about library pools. Second, in parallel, the RNA FASTQ file, the HTO FASTQ file, and, if available, a corresponding Bulk RNA FASTQ file for each sample present in the multiplexed library are provided to a demultiplexing subprocess. The workflow calculates demultiplexing results based on HTO counts, as well as genetic demultiplexing results if the library has corresponding bulk RNA-seq data. Demultiplexing results are stored in all exported SCE objects versions, but libraries themselves are not demultiplexed. Third, only SCE, not AnnData, files are provided for multiplexed libraries.

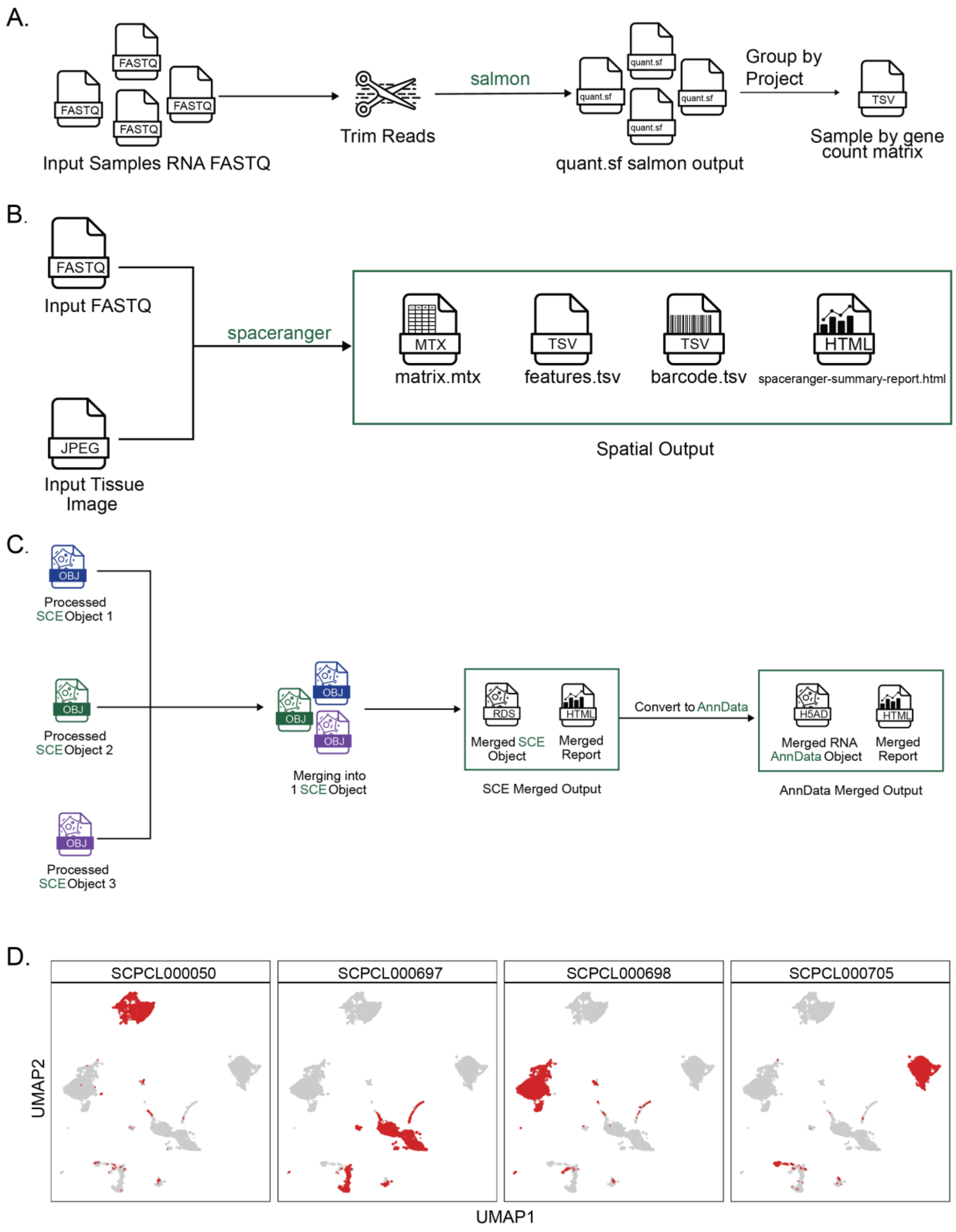

Figure S3: ***Processing other sequencing modalities and merging objects with scpca-nf, Related to Figure 2.***

A. Overview of the bulk RNA-Seq workflow. Reads are trimmed using fastp [S5], and salmon [S6] is used to map reads and quantify counts. Quantified expression files are grouped by project and exported as a sample-by-gene count matrix in TSV format.

B. Overview of the spatial transcriptomics workflow. The FASTQ file and tissue and/or CytAssist image for a given library are input to spaceranger [S7]. spaceranger results are returned without any further processing.

C. Overview of the merged workflow. Processed SCE objects in a given project are merged into a single object, including ADT counts from CITE-seq data if present, and a merged summary report is generated. Merged objects are provided in either SCE or AnnData format.

D. Example of UMAPs as shown in the merged summary report. Cells from the library of interest are in red, and cells from other libraries are in gray. The UMAP was constructed from the merged object with equal library weighting, but no batch correction was performed. The libraries pictured are a subset of libraries in the ScPCA project SCPCP000003. For this figure specifically, the merged UMAP was constructed from these four libraries only, but the merged object and summary report on the ScPCA Portal for SCPCP000003 contain all of this project's libraries.

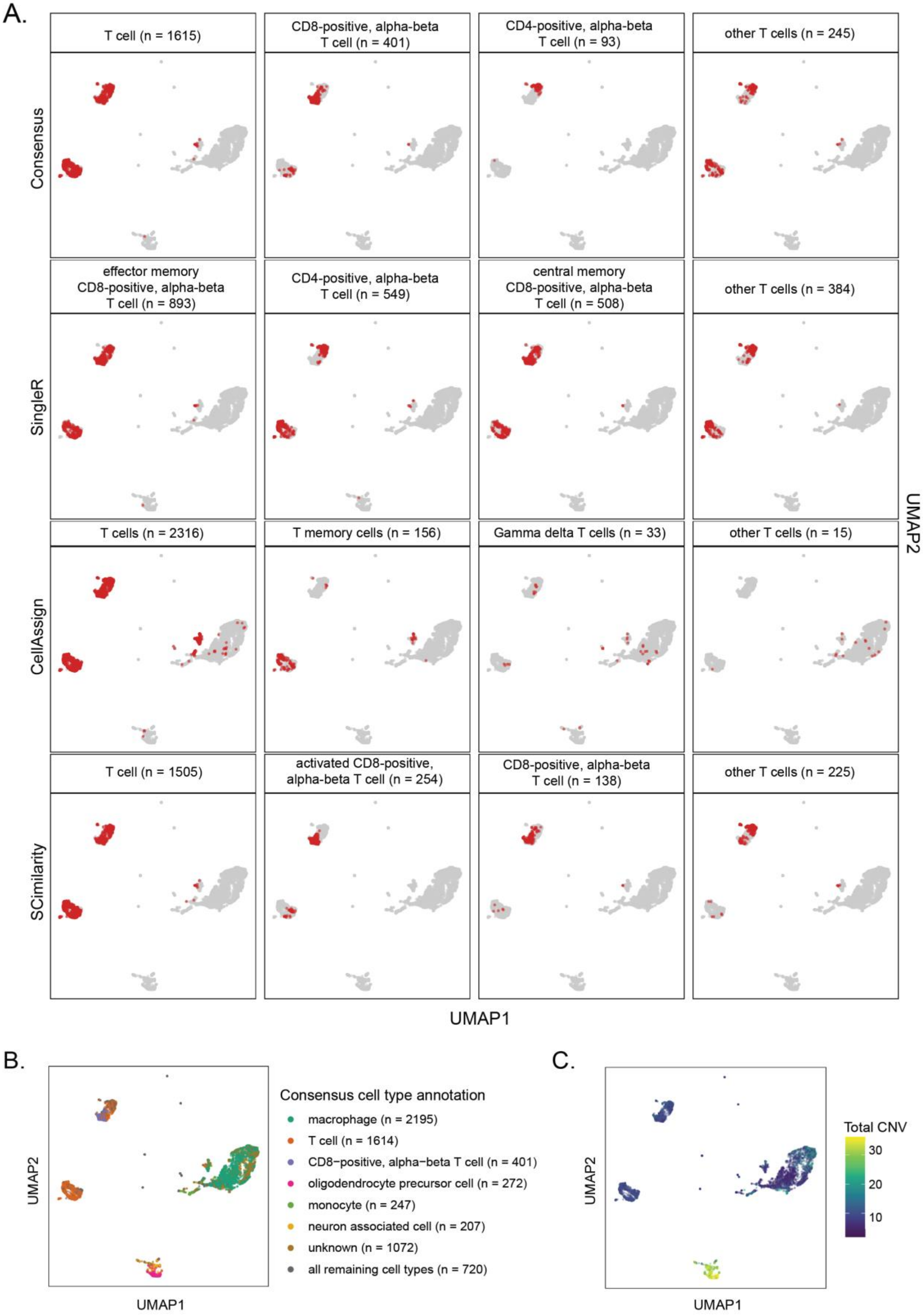
A.
T cell (n = 1615)
CD8-positive, alpha-beta T cell (n = 401)
CD4-positive, alpha-beta T cell (n = 93)
other T cells (n = 245)
Consensus
effector memory CD8-positive, alpha-beta T cell (n = 893)
CD4-positive, alpha-beta T cell (n = 549)
central memory CD8-positive, alpha-beta T cell (n = 508)
other T cells (n = 384)
SingleR
T cells (n = 2316)
T memory cells (n = 156)
Gamma delta T cells (n = 33)
other T cells (n = 15)
CellAssign
T cell (n = 1505)
activated CD8-positive, alpha-beta T cell (n = 254)
CD8-positive, alpha-beta T cell (n = 138)
other T cells (n = 225)
SCimilarity
UMAP1
UMAP2
B.
Consensus cell type annotation
macrophage (n = 2195)
T cell (n = 1614)
CD8−positive, alpha−beta T cell (n = 401)
oligodendrocyte precursor cell (n = 272)
monocyte (n = 247)
neuron associated cell (n = 207)
unknown (n = 1072)
all remaining cell types (n = 720)
UMAP1
UMAP2
C.
Total CNV
30
20
10
UMAP1
UMAP2

Figure S4: ***Ontology-aware consensus cell type assignment provides harmonized labels for cells, Related to Figure 3.***

A. UMAP highlighting cells annotated as types of T cells with SingleR [S8], CellAssign [S9], SCimilarity [S10] as well as the associated consensus cell types for the library SCPCL000049. All other cells are shown in gray. The top three T cell types are shown for each method, with remaining T cell types combined in “other T cells.”

B. UMAP showing the top seven consensus cell types in SCPCL0000049. Other consensus cell types are included in the “all remaining cell types” category.

C. UMAP showing total per-cell CNV events, calculated by summing the number of chromosome arms with a CNV event as estimated by the i6 HMM in InferCNV [S11], for SCPCL0000049.

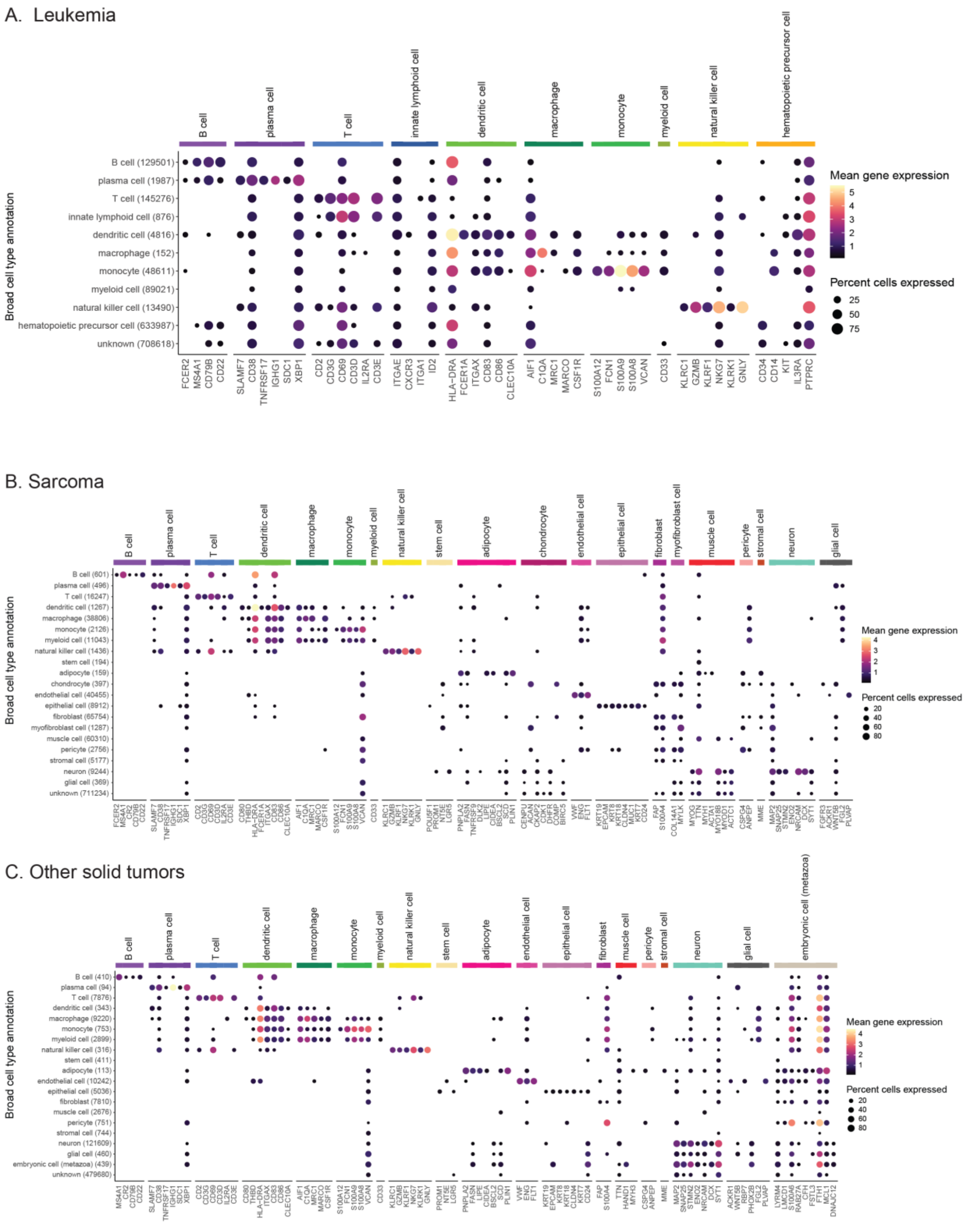

Figure S5: ***Consensus cell type annotation gene expression in other diagnosis groups, Related to Figure 4.***

Dot plots showing expression of cell-type-specific marker genes across libraries from Leukemia (A), Sarcoma (B), and Other solid tumors (C) diagnosis groups. Expression is shown for each broad cell type annotation. The y-axis displays broad consensus cell types. The x-axis displays marker genes, determined by CellMarker 2.0 [S12], used to validate cell types in the top annotation bar. Dots are colored by mean gene expression across libraries and sized proportionally to the percent of libraries they are observed in, out of all cells with the same broad cell type annotation in the given diagnosis. Up to 10 marker genes are shown per broad cell type. Only broad cell type annotations present in at least 50 cells across samples in the given diagnosis group are shown. Cell types without associated marker genes in CellMarker 2.0 are excluded, including pigment cell for Sarcoma (B) and pigment cell and kidney cell for Other solid tumors (C).

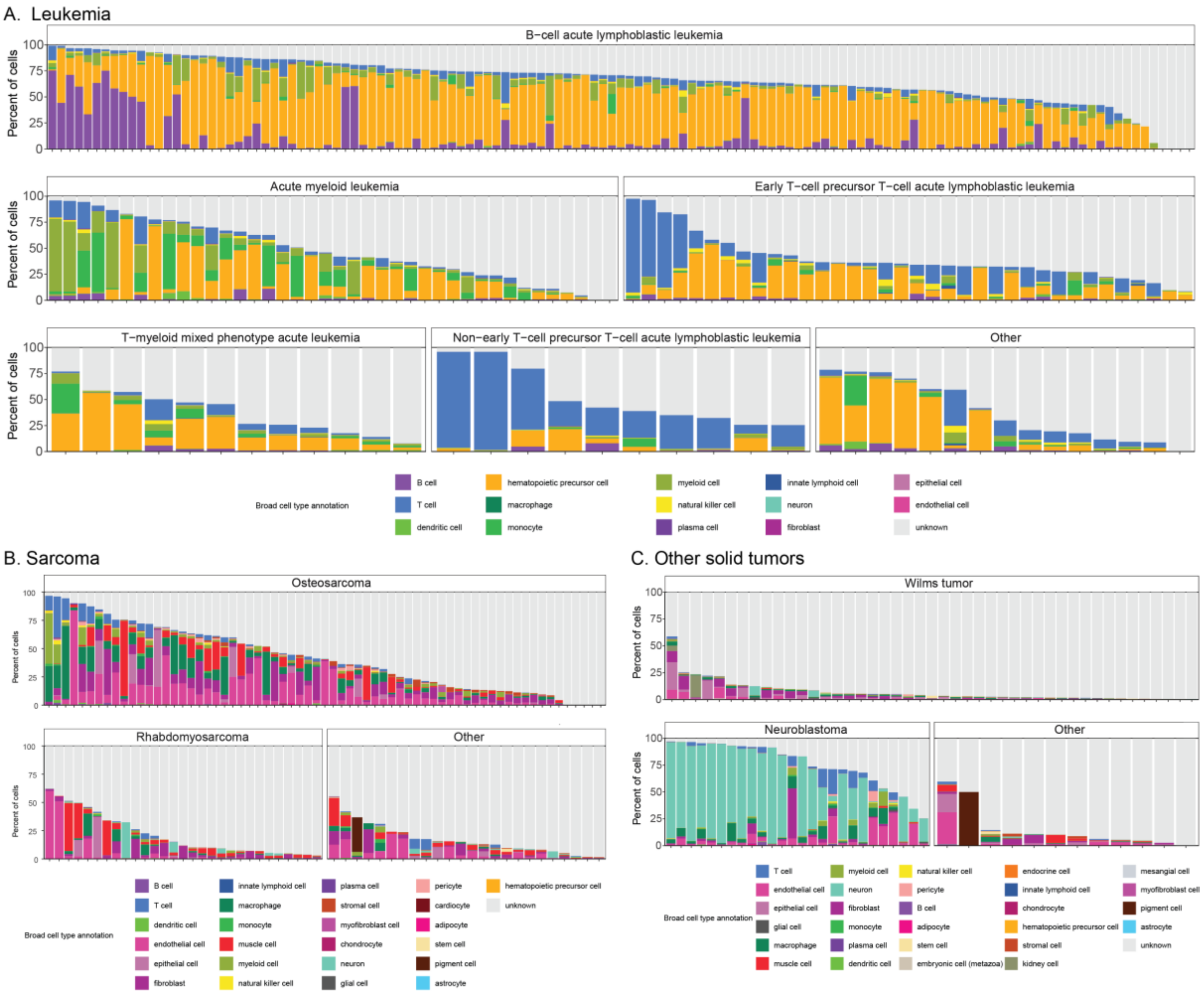

Figure S6: ***Consensus cell type annotation distributions in other diagnosis groups, Related to Figure 4.***

Barplots of the percentage of cells annotated as each broad consensus cell type annotation across all libraries from Leukemia (A), Sarcoma (B), and Other solid tumors (C) diagnosis groups, specifically for non-multiplexed libraries from patient tissue samples Libraries are grouped by diagnosis in each panel. Each column represents the distribution of cell types within a single library.

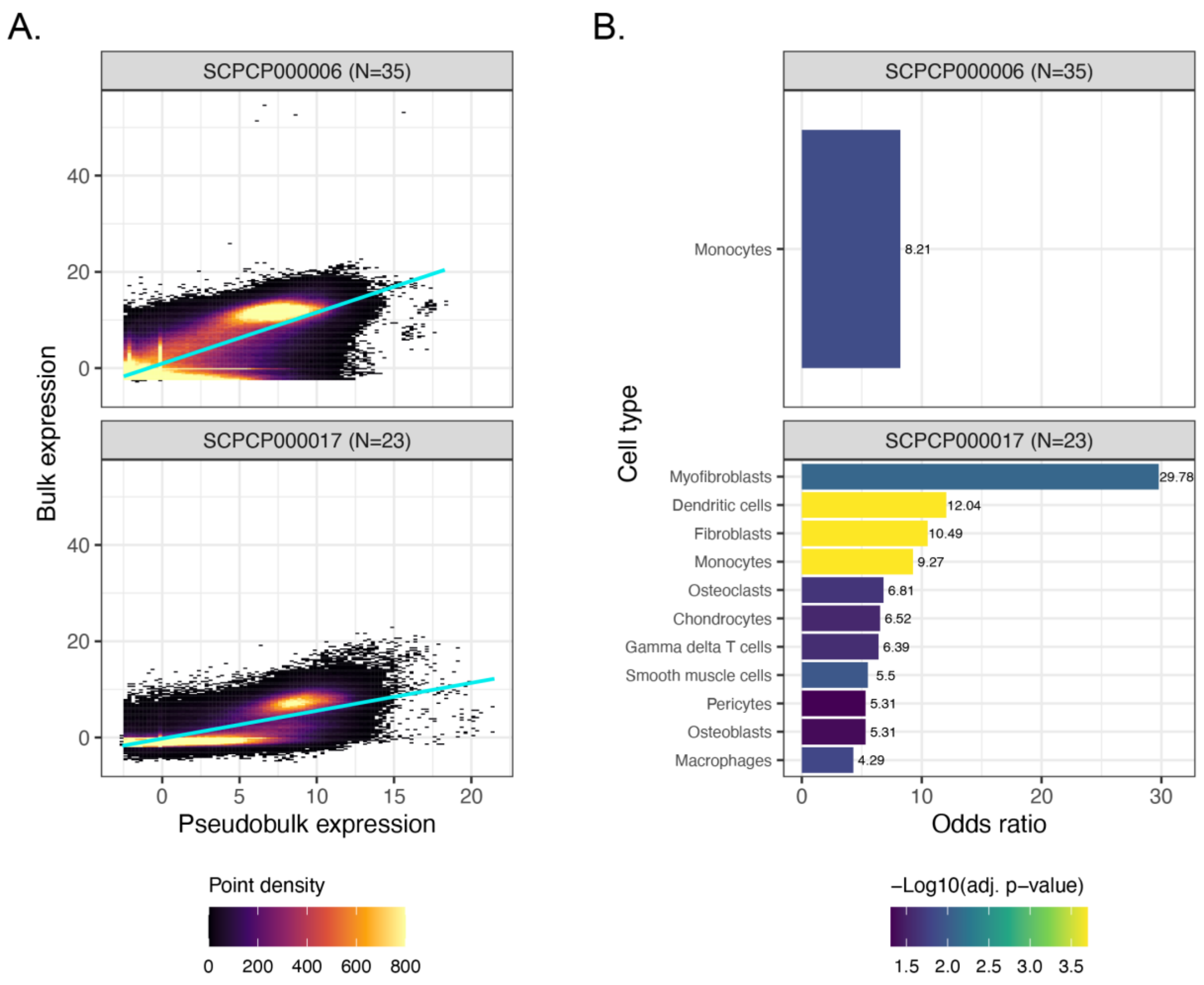

Figure S7: ***Comparison of bulk and pseudobulk modalities for additional projects, Related to Figure 6.***

A. Scatter plots colored by point density of DESeq2-transformed [S13] and normalized bulk RNA-seq expression compared to pseudobulk expression from single-nuclei RNA-seq, with a regression line shown. Projects with RNA-seq for both bulk and single-cell/nuclei modalities that are not displayed in Figure 6A are shown, with sample counts in parentheses. All samples shown here are single-nuclei libraries.

B. Odds ratios, which indicate overrepresentation of cell-type marker genes in bulk relative to single-cell/nuclei RNA-seq, from overrepresentation analysis for the same samples shown in panel A, colored by FDR-corrected significance. 44 cell types were evaluated for project SCPCP000006, and 50 cell types were evaluated for project SCPCP000017.

Supplemental References

S1. He, D., Zakeri, M., Sarkar, H., Soneson, C., Srivastava, A., and Patro, R. (2022). Alevin-fry unlocks rapid, accurate and memory-frugal quantification of single-cell RNA-seq data. Nat Methods *19*, 316–322. 10.1038/s41592-022-01408-3.

S2. 10x Genomics. Cell Ranger v6.1.2. https://www.10xgenomics.com/support/software/cell-ranger/

S3. Zheng, G.X.Y., Terry, J.M., Belgrader, P., Ryvkin, P., Bent, Z.W., Wilson, R., Ziraldo, S.B., Wheeler, T.D., McDermott, G.P., Zhu, J., et al. (2017). Massively parallel digital transcriptional profiling of single cells. *Nat. Commun. 8*, 14049. https://doi.org/10.1038/ncomms14049

S4. Virshup, I., Rybakov, S., Theis, F.J., Angerer, P., and Wolf, F.A. (2024). anndata: Access and store annotated data matrices. JOSS *9*, 4371. 10.21105/joss.04371.

S5. Chen, S., Zhou, Y., Chen, Y., and Gu, J. (2018). fastp: an ultra-fast all-in-one FASTQ preprocessor. Bioinformatics *34*, i884–i890. 10.1093/bioinformatics/bty560.

S6. Patro, R., Duggal, G., Love, M.I., Irizarry, R.A., and Kingsford, C. (2017). Salmon provides fast and bias-aware quantification of transcript expression. Nat Methods *14*, 417–419. 10.1038/nmeth.4197.

S7. 10x Genomics. Space Ranger. https://www.10xgenomics.com/support/software/space-ranger/

S8. Aran, D., Looney, A.P., Liu, L., Wu, E., Fong, V., Hsu, A., Chak, S., Naikawadi, R.P., Wolters, P.J., Abate, A.R., et al. (2019). Reference-based analysis of lung single-cell sequencing reveals a transitional profibrotic macrophage. Nat Immunol *20*, 163–172. 10.1038/s41590-018-0276-y.

S9. Zhang, A.W., O'Flanagan, C., Chavez, E.A., Lim, J.L.P., Ceglia, N., McPherson, A., Wiens, M., Walters, P., Chan, T., Hewitson, B., et al. (2019). Probabilistic cell-type assignment of single-cell RNA-seq for tumor microenvironment profiling. Nat Methods *16*, 1007–1015. 10.1038/s41592-019-0529-1.

S10. Heimberg, G., Kuo, T., DePianto, D.J., Salem, O., Heigl, T., Diamant, N., Scalia, G., Biancalani, T., Turley, S.J., Rock, J.R., et al. (2024). A cell atlas foundation model for scalable search of similar human cells. Nature *638*, 1085–1094. 10.1038/s41586-024-08411-y.

S11. Broad Institute. InferCNV: Inferring Copy Number Alterations from Tumor Single Cell RNA-Seq Data. https://github.com/broadinstitute/inferCNV.

S12. Hu, C., Li, T., Xu, Y., Zhang, X., Li, F., Bai, J., Chen, J., Jiang, W., Yang, K., Ou, Q., et al. (2022). CellMarker 2.0: an updated database of manually curated cell markers in human/mouse and web tools based on scRNA-seq data. Nucleic Acids Research *51*, D870–D876. 10.1093/nar/gkac947.

S13. Love, M.I., Huber, W., and Anders, S. (2014). Moderated estimation of fold change and dispersion for RNA-seq data with DESeq2. Genome Biol *15*, 550. 10.1186/s13059-014-0550-8.
