# Supplementary material for: The Single-Cell Pediatric Cancer Atlas: Data portal and open-source tools for single-cell transcriptomics of pediatric tumors
Source: Cell Genom. 2026 Jun 24;6(7):101283. doi: 10.1016/j.xgen.2026.101283 (PMC13347941; doi:10.1016/j.xgen.2026.101283)
Supplement: Document S2. Transparent peer review records for Hawkins et al [file mmc4.pdf]

Summary

Initial submission: Received: 5/29/2025

Scientific editor: Laura Zahn

First round of review: Number of reviewers:
Revision invited: 9/18/2025
Revision received: 2/12/2026

Second round of review: Number of reviewers:
Accepted: 5/28/2026

Data freely available: Yes

Code freely available: Yes

This transparent peer review record is not systematically proofread, type-set, or edited. Special characters, formatting, and equations may fail to render properly. Standard procedural text within the editor's letters has been deleted for the sake of brevity, but all official correspondence specific to the manuscript has been preserved.

Referees' reports, first round of review

Reviewer #1: In this manuscript that accompanies the Ales's Lemonade Stand ScPCA Portal, a massive collection of pediatric oncology single cell transcriptomic datasets, the authors describe the presentation of, and steps taken to standardize processing of these data. They provide a standardized framework for processing of single cell and single nuclei RNAseq data that is independent of 10X Cell Ranger. This is an extremely valuable resource for the entire pediatric oncology community.

My main concern is that there is no provision for non-bioinformatic browsing of the data in the scPCA portal, such as that provided by the UCSC portal (https://cells.ucsc.edu). A browsable view of the data would greatly improve the usability of this resource by the broader research community, as it is currently only usable by those with programming experience. Indeed, the entire manuscript is largely tailored to bioinformatics readers.

A minor issue, as noted by the authors, is the use of reference sets of normal cell types to annotate neoplastic cell types. The non-neoplastic populations, largely immune, are more likely to be accurately annotated than neoplastic clusters. An example of the inaccuracy of using normal cell references is apparent in two samples that I downloaded (an anaplastic glioma and an ependymoma). In both cases the predominant neoplastic cluster was annotated as "neuron". Hopefully as the portal evolves consensus

pediatric oncology neoplastic cell type annotations could be incorporated using their multiple classifier consensus approach as discussed in Figure 5.

Overall, I feel this manuscript is worthy of publication but could be of far broader usability with the addition of a UCSC type browser.

Reviewer #2: Hawkins and colleagues present The Single-cell Pediatric Cancer Atlas (ScPCA), a freely accessible central resource for uniformly processed single-cell/nuclei data from pediatric tumor samples. The data in total includes 10 projects with over 700 samples across 55 cancer entities. Although there exist a number of single-cell data repositories, none of those has a clear focus on pediatric cancers. In contrast to adult cancers, public available single-cell data for pediatric cancers is underrepresented. This makes this resource (ScPCA) extremely valuable for this research field. Moreover, the authors provide a streamlined nextflow pipeline to, not only reproduce the results, but also to run the same uniform processing on own data to facilitate comparison with data in the atlas or to prepare the data for submission.

Strengths:
- clean, efficient and well documented processing pipeline
- freely available and transparent data and code
- clean and well structured organization of the data
- data downloadable in different formats (.h5ad, .rds) which gives support to users of both universes R and Python
- data available in different processing stages: unprocessed, filtered, processed. This gives the user more freedom on how to further use the data.
- project data downloadable as single samples or as merged sample
- rich and consistent/harmonized metadata
- automatic cell type annotation but with retaining also original labels in case they are present
- integrated support for multiple modalities and bulk RNAseq data
- open for submission
- very well written manuscript

In summary, I want to congratulate and thank the authors for this nice and clean work.

Minor comments and suggestions:

- The portal would benefit from an API (python/R/REST) for searching, selecting and downloading.
- I'd strongly suggest to also integrate scimilarity for automated AI based cell type annotation.
- The authors should consider to store multimodal data in mudata objects and saving them as .h5mu

- Comparison cellranger with alevin-fry (Figure S1A):
The authors should explain in the paper why the R values for the snRNA-seq samples are constantly higher than the ones for the scRNA-seq samples. It would also be of interest to know on which basis the samples for the comparison were selected, was it random choice or subjective.

- Multiplexed libraries:
"To process multiplexed libraries, the HTO FASTQ les are quanti ed using salmon alevin andalevin-fry (Figure S2C)."
I guess the authors are referring to Figure S2E

- Figure S2B,C: the plots are missing the y-scale, please add.
- Figure S2D: the color bar is missing

- Overall the manuscript might be shortened in some points where it is redundant/repetitive.

Reviewer #3: The authors present a resource comprising 700 samples from various pediatric tumors,

uniformly processed and analyzed. While the dataset has the potential to be valuable for the community, several aspects limit its overall impact and usability and need to be addressed to strengthen the work.
Major Points :

1. The manuscript provides multiple processed files for download but does not include raw FASTQ data or comprehensive patient-level metadata, such as genomic mutations, translocations, tumor cell fractions, diagnostic criteria, whole-genome or exome data, and detailed cancer subtypes (e.g., leukemia classifications). These are essential for reproducibility, independent validation, and maximizing the utility of the dataset. Making these datasets available would substantially enhance the value of this resource.

2. The rationale for developing and using the scpca-nf workflow is unclear, given that robust and widely adopted tools such as Seurat and Scanpy already exist. Benchmarking scpca-nf against these tools and demonstrating advantages in accuracy, scalability, reproducibility, or usability would help justify its development.

3. The resource requires users to have coding expertise, limiting accessibility for many biomedical researchers. A graphical interface or web portal would significantly improve usability and encourage broader adoption.

4. The integration of datasets and handling of batch effects require further clarification. The manuscript does not describe in detail how datasets from different laboratories, sequencing platforms, and assay versions were merged or how batch effects were assessed and corrected. Dimensionality reduction plots, such as UMAPs, should be presented before and after integration to show whether batch effects were adequately addressed.

5. The uniformity of cell type annotations across datasets is not demonstrated. Because marker selection and naming conventions can vary across groups, it is important to provide evidence (such as marker gene expression plots or heatmaps) to confirm consistency, along with manual curation for functionally important subsets such as exhausted, central memory, effector memory, and naïve T cells.

6. The approach used to distinguish malignant from normal cells, particularly in diseases like AML, is not clearly described. Methods such as InferCNV or mutation mapping should be applied and representative results included to confirm tumor cell labeling.

7. The manuscript does not specify how doublets were identified and removed or their prevalence in the datasets. The treatment of antibody-derived tag (ADT) data would also benefit from more detail on how background hybridization was subtracted and how normalization was performed.

8. Although the resource includes functionality to merge datasets, it lacks batch correction utilities. Given the diversity of data sources, either batch effects should be corrected directly or it should be demonstrated that they do not materially affect the data.

9. The manuscript focuses largely on describing the dataset and analysis workflow but provides minimal biological interpretation or translational insight. Including exemplar analyses of tumor, immune, and stromal compartments to identify biologically or clinically relevant features—such as outcome-associated signatures or novel cell states—would help demonstrate the resource's utility and better justify publication.

10, The manuscript repeatedly refers to the resource as "open source," but without raw FASTQ data and full metadata, this description is misleading. The scope of "open source" and any plans for raw data release should be clarified. Raw data must be made available

11. Figure 6 shows only broad cell lineages that can be reproduced by standard workflows. Including higher-resolution annotations (e.g., T cell subsets and tumor subpopulations) would make it more informative.

12. Clarify whether the resource provides batch correction tools in addition to merging functionality, or state explicitly that users must handle corrections themselves.
13. Ensure that methods for doublet detection, ADT background subtraction, and normalization are described clearly in the methods section for transparency and reproducibility.

Overall, while this dataset has the potential to serve as a valuable community resource for pediatric tumor research, the current manuscript falls short in several critical areas, including data accessibility, usability, methodological transparency, and biological interpretation. Addressing these issues, by providing raw data

and metadata, ensuring proper batch correction and annotation, improving user accessibility, and including analyses that yield meaningful biological or clinical insights would substantially enhance the impact and value of this work.

Authors’ response to the first round of review

Reviewer #1: In this manuscript that accompanies the Ales's Lemonade Stand ScPCA Portal, a massive collection of pediatric oncology single cell transcriptomic datasets, the authors describe the presentation of, and steps taken to standardize processing of these data. They provide a standardized framework for processing of single cell and single nuclei RNAseq data that is independent of 10X Cell Ranger. This is an extremely valuable resource for the entire pediatric oncology community.

My main concern is that there is no provision for non-bioinformatic browsing of the data in the scPCA portal, such as that provided by the UCSC portal (https://cells.ucsc.edu). A browsable view of the data would greatly improve the usability of this resource by the broader research community, as it is currently only usable by those with programming experience. Indeed, the entire manuscript is largely tailored to bioinformatics readers.

We agree and thank the reviewer for pointing this limitation out. To address this comment and enable no-code browsing of the ScPCA data, we have implemented a standalone version of the UCSC Cell Browser that can be accessed via the "Visualize" tab in the navigation bar of the ScPCA Portal. The Cell Browser interface is now available here: https://scpca.alexslemonade.org/visualize.

The screenshot shown in Figure 1C has been updated to show an example of browsing cell type annotations from a single sample with the "Visualize" tab.

A minor issue, as noted by the authors, is the use of reference sets of normal cell types to annotate neoplastic cell types. The non-neoplastic populations, largely immune, are more likely to be accurately annotated than neoplastic clusters. An example of the inaccuracy of using normal cell references is apparent in two samples that I downloaded (an anaplastic glioma and an ependymoma). In both cases the predominant neoplastic cluster was annotated as "neuron". Hopefully as the portal evolves consensus pediatric oncology neoplastic cell type annotations could be incorporated using their multiple classifier consensus approach as discussed in Figure 5.

We agree entirely that the automated, reference-based methods we use have considerable limitations when applied to pediatric neoplastic cells.

In part to address these limitations, we launched a project to openly analyze the ScPCA data called OpenScPCA in 2024 (https://openscpca.readthedocs.io/en/latest/) with one goal being to expertly or “more manually” label cell types, including the identification of malignant cells and cell states. As part of our dedication to this effort, we made ALSF grants available to support completion of cell type labeling (https://github.com/AlexsLemonade/OpenScPCA-analysis/discussions/1062). Labeling cell types as part of OpenScPCA guarantees transparency and extensibility, as all code is publicly available, openly licensed, and peer reviewed.

Response to Reviewers

As part of this revision, we have integrated trusted OpenScPCA-derived cell type labels into the data downloads available from the Portal, specifically for these two projects: SCPCP000015 (Ewing's sarcoma; analytical code here: https://github.com/AlexsLemonade/OpenScPCA-analysis/tree/main/analyses/cell-type-ewings) and SCPCP000004 (Neuroblastoma; analytical code that makes use of NBAtlas [Bonine et al. 2024.] here: https://github.com/AlexsLemonade/OpenScPCA-analysis/tree/main/analyses/cell-type-neuroblastoma-04).

We have further updated the cell type HTML reports available for download from the Portal to include these cell type labels for relevant libraries. We have additionally added a new Figure 5 to the manuscript, which includes the results from OpenScPCA annotations for project SCPCP000004 (neuroblastoma). Moving forward, we are committed to continuing to improve neoplastic cell labeling by annotating more projects through OpenScPCA and adding results to the Portal.

Although we do not explicitly annotate neoplastic cells for all projects on the Portal, we have added copy-number variation estimates from InferCNV for samples with sufficient non-neoplastic cells to serve as a reference as part of this revision. These results can be combined with consensus cell types to aid in the annotation of neoplastic cells. An example of this is shown in Figure 5D.

Overall, I feel this manuscript is worthy of publication but could be of far broader usability with the addition of a UCSC type browser.

Reviewer #2: Hawkins and colleagues present The Single-cell Pediatric Cancer Atlas (ScPCA), a freely accessible central resource for uniformly processed single-cell/nuclei data from pediatric tumor samples.

The data in total includes 10 projects with over 700 samples across 55 cancer entities. Although there exist a number of single-cell data repositories, none of those has a clear focus on pediatric cancers. In contrast to adult cancers, public available single-cell data for pediatric cancers is underrepresented. This makes this resource (ScPCA) extremely valuable for this research field. Moreover, the authors provide a streamlined nextflow pipeline to, not only reproduce the results,
but also to run the same uniform processing on own data to facilitate comparison with data in the
atlas or to prepare the data for submission.

Strengths:

- clean, efficient and well documented processing pipeline
- freely available and transparent data and code
- clean and well structured organization of the data
- data downloadable in different formats (.h5ad, .rds) which gives support to users of both universes R and Python
- data available in different processing stages: unprocessed, filtered, processed. This gives the user more freedom on how to further use the data.
- project data downloadable as single samples or as merged sample
- rich and consistent/harmonized metadata
- automatic cell type annotation but with retaining also original labels in case they are present

- integrated support for multiple modalities and bulk RNAseq data
- open for submission
- very well written manuscript

In summary, I want to congratulate and thank the authors for this nice and clean work.

We sincerely appreciate the reviewer's kind words.

Minor comments and suggestions:

- The portal would benefit from an API (python/R/REST) for searching, selecting and downloading.

We thank the reviewer for this helpful suggestion. As part of the revision, we have written a new open-source R package called ScPCAr (github.com/AlexsLemonade/ScPCAr/) that leverages the R package httr2 to support programmatic access to data and metadata from the Portal. We now describe this package in the manuscript (lines 281-283):

"In addition to the web interface, we provide an R package, ScPCAr, to allow programmatic access to metadata and files on the Portal, available at https://alexslemonade.github.io/ScPCAr."

We have also added more documentation to our API and linked to it in the manuscript (lines 847-849):

"Data can be downloaded both via the Portal web interface and programmatically using the ScPCAr R package, which is a wrapper for the ScPCA Portal API (https://api.scpca.alexslemonade.org/docs/)."

- I'd strongly suggest to also integrate scimilarity for automated AI based cell type annotation.

We agree that SCimilarity (Heimberg et al. 2024.) would be an excellent addition. Thank you for this suggestion. We have added SCimilarity to scpca-nf, the downloadable objects available from the Portal, and consensus cell type calling. In our updated consensus cell type labeling approach, a consensus cell type is assigned when two out of three methods (SingleR, CellAssign, SCimilarity) agree. Through this method, we have increased the number of cells with a consensus label.

- The authors should consider to store multimodal data in mudata objects and saving them as .h5mu

Thank you for this future-looking suggestion. We have included instructions for converting from multiple AnnData objects to a MuData object in the Getting Started section of our documentation to support users who want to work with MuData objects. This documentation is now available here:
https://scpca.readthedocs.io/en/stable/faq.html#what-if-i-want-to-use-mudata-instead-of-anndat
a-objects

As noted in our responses to Reviewers 1 and 3, we have also added UCSC Cell Browser functionality to the ScPCA Portal, which requires that RNA data be stored as separate AnnData files. We also want to maintain compatibility with CELLxGENE, which we do not believe supports MuData yet (docs). We will certainly consider adding this functionality to scpca-nf in the future as the format grows in popularity/support.

- Comparison cellranger with alevin-fry (Figure S1A):

The authors should explain in the paper why the R values for the snRNA-seq samples are constantly higher than the ones for the scRNA-seq samples. It would also

be of interest to know on which basis the samples for the comparison were selected, was it random choice or subjective.

Thank you for bringing up these important points, and we apologize for not including more information about benchmarking at the time of submission. The libraries shown were randomly chosen from the submitted libraries.

At the time of benchmarking, the latest version of Cell Ranger available was v6.1.2, which recommended using the --include_introns flag to include intronic reads during alignment for single-nucleus libraries only, not single-cell libraries. The index we used for all single-cell and single-nuclei libraries with salmon alevin and alevin-fry contains both spliced and unspliced reads, as recommended by the authors in their publication (He et al. 2022). Because of slight differences in the index used for single-cell versus single-nuclei libraries, the R2 values for single-cell libraries are expected to be slightly lower than those for single-nuclei libraries. Notably, as of Cell Ranger v7.0.0, intronic reads are included by default for all libraries, mirroring the index that we use with salmon alevin and alevin-fry for all libraries on the Portal.

To clarify these points, we have updated the manuscript to include the following text in the legend for Figure S1 (lines 1124 - 1129):

"Results shown were generated with CellRanger v6.1.2 using default parameters for single-cell libraries and use of the --include_introns flag to include intronic reads for single-nuclei libraries only. All libraries were processed with salmon alevin v1.5.2 and alevin-fry v0.4.1 using an index containing both spliced and unspliced cDNA as mentioned in the Methods. The libraries used for benchmarking were randomly chosen."

- Multiplexed libraries:

"To process multiplexed libraries, the HTO FASTQ les are quanti ed using salmon alevin andalevin-fry (Figure S2C)."

I guess the authors are referring to Figure S2E

- Figure S2B,C: the plots are missing the y-scale, please add.
- Figure S2D: the color bar is missing

Thank you for catching these errors and bringing them to our attention. We have resolved these errors as part of the revision.

- Overall the manuscript might be shortened in some points where it is redundant/repetitive.

We have taken this opportunity to remove the repetition the reviewer thoughtfully suggested.

Reviewer #3: The authors present a resource comprising 700 samples from various pediatric tumors, uniformly processed and analyzed. While the dataset has the potential to be valuable for the community, several aspects limit its overall impact and usability and need to be addressed to strengthen the work.

Major Points :

1. The manuscript provides multiple processed files for download but does not include raw FASTQ data or comprehensive patient-level metadata, such as genomic mutations, translocations, tumor cell fractions, diagnostic criteria, whole-genome or exome data, and detailed cancer subtypes (e.g., leukemia classifications). These are essential for reproducibility, independent validation, and maximizing the utility of the dataset. Making these datasets available would substantially enhance the value of this resource.

The Single-cell Pediatric Cancer Atlas (ScPCA) Portal is an open-access resource; data is freely available for download upon agreement to the Terms of Use, without requiring controlled

access, such as would be granted by a Data Access Committee. Thus, it is crucial to balance researchers' convenience with the protection of human subjects' privacy, and it is not appropriate to distribute raw, sequence-level patient data via the Portal. However, all ALSF-funded projects must make the raw data available in an appropriate repository, such as dbGaP, and we include links to those data on the Portal as they become available. Furthermore, in many cases, we received, harmonized, and made available as much patient-level metadata as ethics board review permitted.

Many projects have sample-level metadata beyond the fields that we mapped to ontology terms (i.e., those in Table 1: age, sex, organism, diagnosis, tissue of origin, and ethnicity [if applicable]): disease timing (present in 100% of projects) and subdiagnosis (present in 47.8% of projects and which often contains more detailed subtype information). In addition, 82.6% of projects have metadata beyond the base-level set of fields we attempted to collect for all projects. For example, the project SCPCP000008, which comprises acute lymphoblastic leukemia and mixed phenotype acute leukemia samples, includes subtype information in the subdiagnosis field (e.g., TCF3::PBX1, BCR::ABL1), karyotype, blast percentage, and risk group. SCPCP000010, which is primarily composed of low-grade glioma and atypical teratoid rhabdoid tumor samples, includes the last known clinical status and information about treatment (e.g., chemotherapy status, extent of tumor resection). The neuroblastoma project SCPCP000004 includes information on MYCN amplification and on treated vs. nontreated samples. We highlight this subset of projects and sample-level metadata fields to demonstrate that many projects have more comprehensive metadata than the manuscript alone may suggest.

To clarify the availability of additional metadata, the manuscript now reads (changes in bold):

In the Results section (lines 144 - 147): "All samples on the Portal include a core set of metadata obtained from investigators, including age, sex, diagnosis, subdiagnosis (if applicable), tissue location, and disease stage. The majority of projects include additional metadata, such as treatment or tumor stage, if provided by submitters. We standardized all provided metadata to maintain consistency across projects before adding it to the Portal."

In the Methods section on Metadata (lines 489 - 491): "The majority (82.6%) of projects on the Portal have additional metadata fields, such as the presence or absence of treatment, tumor grade, or whether a sample was obtained from a primary tumor or metastasis."

2. The rationale for developing and using the scpca-nf workflow is unclear, given that robust and widely adopted tools such as Seurat and Scanpy already exist. Benchmarking scpca-nf against these tools and demonstrating advantages in accuracy, scalability, reproducibility, or usability would help justify its development.

We thank the reviewer for the opportunity to clarify the rationale for developing scpca-nf. scpca-nf does not "compete" with widely adopted frameworks such as Seurat and Scanpy, which typically perform filtering, normalization, and dimensionality reduction. Instead, the Nextflow workflow can be thought of as a convenient "wrapper" and coordinator for all steps required for processing sc/snRNA-seq (and other supported modalities): quantification, filtering, normalization, clustering, dimensionality reduction, and conversion to widely used formats. We demonstrate that our choice of quantification tool (i.e., alevin-fry) yields lower computational costs (i.e., memory usage and runtime) than Cell Ranger, with similar quantification results (Figure S1).

Using a workflow management system, such as Nextflow, allows for portability, reproducibility,

and parallelization beyond what can be achieved with, e.g., shell scripting alone (Langer et al. 2025.). Indeed, we have accepted community-contributed datasets processed on multiple institutions' computing infrastructure, demonstrating the portability and usability of scpca-nf. From January 15-29, 2026, GitHub reported 807 clones of the workflow by 192 unique cloners, further suggesting the usage of scpca-nf. All steps within scpca-nf are containerized, supporting reproducibility.

Regarding methods or frameworks that "compete" with Seurat and Scanpy that are included in scpca-nf, we elected to use methods within the Bioconductor ecosystem that operate on SingleCellExperiment objects. We made this design choice for several reasons. We expected more R users than Python users (as borne out by our usage data: RDS downloads were generated ~2.7x more often than AnnData downloads from mid-December 2024 through August 2025), which led us to develop within the R ecosystem. More thorough documentation, a stable object format, and smaller file sizes in the Bioconductor ecosystem, compared with Seurat, benefit us as developers and our users.

To make the benefit of using Nextflow clearer, we have added the following to the Results section of the manuscript (lines 194 - 197):

"Nextflow is a workflow management system that allows users to execute multi-step and long-running bioinformatics processes in a portable and reproducible manner across various computing environments, including high-performance computing clusters and cloud-based computing (Langer et al. 2025.)."

Regarding the use of Bioconductor, we have added the following to the Results section (lines 218 - 220):

"We elected to use the Bioconductor ecosystem (Gentleman et al. 2004.; Huber et al. 2015.) for filtering, normalization, and dimensionality reduction because of its rich documentation, wide use in the community, and ability to produce relatively small file sizes."

3. The resource requires users to have coding expertise, limiting accessibility for many biomedical researchers. A graphical interface or web portal would significantly improve usability and encourage broader adoption.

We agree that the current implementation imposes barriers to adoption for researchers with limited coding experience. To address this, we have implemented a standalone version of the UCSC Cell Browser, accessible via the "Visualize" tab in the navigation bar of the ScPCA Portal: https://scpca.alexslemonade.org/visualize

4. The integration of datasets and handling of batch effects require further clarification. The manuscript does not describe in detail how datasets from different laboratories, sequencing platforms, and assay versions were merged or how batch effects were assessed and corrected. Dimensionality reduction plots, such as UMAPs, should be presented before and after integration to show whether batch effects were adequately addressed.

No integration of datasets and handling of batch effects was performed. In the methods section on Generating merged data (lines 741 - 742), we state, "The merge.nf workflow does not perform batch correction or integration, so the counts in the merged object are not batch-corrected."

We have added the following sentence to the Results section on Merged objects (lines 304 - 305):

"Merged objects are not batch-corrected or integrated, so users can perform their own batch

correction or integration as needed to suit their experimental designs."

5. The uniformity of cell type annotations across datasets is not demonstrated. Because marker selection and naming conventions can vary across groups, it is important to provide evidence (such as marker gene expression plots or heatmaps) to confirm consistency, along with manual curation for functionally important subsets such as exhausted, central memory, effector memory, and naïve T cells.

We agree that marker gene selection and naming conventions varying across research groups can be a barrier to uniformity. As such, we have enforced uniformity in our cell type annotations across samples by using two reference-based automated methods (i.e., SingleR and CellAssign) with a common set of references in which cell types have been mapped to the Cell Ontology. As part of this revision, we have additionally included the automated method SCimilarity, which uses a foundation model to assign cell types that have also been mapped to the Cell Ontology. We further identify consensus among the SingleR, CellAssign, and SCimilarity calls using an ontology-aware approach. Therefore, heterogeneity in annotation methods among research groups has been avoided in our procedure.

Dot plots of marker gene expression, where marker genes were selected using CellMarker 2.0 as described in the Methods, for the consensus cell label calls are present in Figures 4A and S5A-C and generally show consistency between the consensus cell type label and marker gene expression. Note that these figures show broad cell types to conserve space.

Per the reviewer's suggestion, we now also show a finer-granularity immune cell dot plot in Figure 4D, which provides an immune-specific complement to the broad cell types shown in Figure 4A.

Our goal in providing uniform, consensus cell type annotations is to save users of the Portal time in completing their downstream analyses. CellAssign, in particular, takes multiple days to run on all samples included in the Portal. By providing consensus cell type labels for normal cells, we allow ScPCA users to focus on characterizing "unknown" cell types that are expected to be malignant in ways that suit their own purposes and experimental designs. The idea of using labels when there is no or low consensus to prioritize manual study/follow-up and "reduce the load of manual inspection" comes from Ergen et al. (2024).

6. The approach used to distinguish malignant from normal cells, particularly in diseases like AML, is not clearly described. Methods such as InferCNV or mutation mapping should be applied and representative results included to confirm tumor cell labeling.

We thank the reviewer for pointing out this important aspect of cell type identification. As the reviewer implies, it is difficult to consistently and automatically distinguish malignant from normal cells across the 55 cancer types in the ScPCA resource.

Per the reviewer's suggestion, we have now implemented a step in the scpca-nf workflow to apply InferCNV to samples that have enough cells to serve as a reference (i.e., that are predicted to be normal through our consensus cell type calling approach) and report the results in objects and a summary in the cell type report that can be downloaded from the Portal. We now describe this procedure in the manuscript, and we have added several figures showcasing results from running InferCNV (Figure 5B-D for results from neuroblastoma libraries, and Figure S4C for results from a ganglioglioma library). We anticipate that this addition will facilitate

downstream users' classification of malignant cells.
As stated above in our response to Reviewer 1, we have also ported "more manual" annotations of malignant cells from the OpenScPCA project (https://openscpca.readthedocs.io/en/latest/) into objects that will be available for download from the Portal, and we have incorporated this information into the cell type report for select projects. We have specifically ported these labels for projects SCPCP000004 (neuroblastoma) and SCPCP000015 (Ewing sarcoma). Using labels from the OpenScPCA project ensures transparency into how, for example, marker genes are selected, because all code is publicly available and peer-reviewed. We also include an example illustrating the utility of these labels, using libraries from SCPCP000004 (see Figure 5).
7. The manuscript does not specify how doublets were identified and removed or their prevalence in the datasets. The treatment of antibody-derived tag (ADT) data would also benefit from more detail on how background hybridization was subtracted and how normalization was performed.
No doublet detection or removal was performed previously, and we thank the reviewer for raising this point. We have added the results of the doublet detection method scDblFinder to the filtered and processed objects that will be available from the Portal for download to facilitate downstream users' removal of doublets. We have additionally added the following sentence to the Methods section on doublet detection (lines 529 - 534):
"These filtered objects additionally contain results from doublet detection performed with scDblFinder::scDblFinder() (Germain et al. 2021.), including each cell's predicted class ("singlet" or "doublet") as well as the associated doublet score. However, predicted doublets were not filtered out; users can instead use these scDblFinder results to filter doublets as needed for their specific analysis needs."
Regarding ADT processing, we did not attempt to subtract the background levels in our processing pipeline. This is because we prefer to leave this step to the user's discretion for their given analysis. Several methods exist for denoising ADT data, each with different assumptions as well as sources of input information; some leverage information from isotype controls (Mulè et al. 2022.), some consider a global ambient profile in calculations (Mulè et al. 2022.; Fleming et al. 2023.), some use only data-driven thresholds (Oliaeimotlagh et al. 2025.), and some do not employ a data-driven approach at all (Stuart et al. 2019.). In addition, any analysis that removes background expression also has the potential to remove meaningful biological signals that may be relevant to the scientific question being asked (Fleming et al. 2023.). As such, we believe our users can achieve more robust background subtraction by choosing a method that is well-suited to the ScPCA samples they analyze and to their scientific goals.
We have therefore added this sentence to clarify that we did not perform background subtraction (lines 590 - 593):
"Additionally, note that we did not perform background subtraction on the ADT counts, but we provide the ambient profile calculated with DropletUtils::ambientProfileEmpty(), which users can employ to perform global de-noising as needed."
In addition, we have updated the text in the Methods section to clarify our approach to ADT normalization as follows, where added information is shown in bold (lines 580 - 587):
"ADT count data were then normalized using scuttle::computeMedianFactors(), which calculates a per-cell size factor as the median ratio of the cell's counts to the background profile previously calculated with DropletUtils::ambientProfileEmpty(). We then

used these factors to normalize ADT counts with scuttle::logNormCounts(). If median-based normalization failed for any reason, ADT counts were log-transformed after adding a pseudocount of 1. We only performed normalization on cells that would be retained after ADT filtering; we assigned NA normalized counts to any cells that would be filtered out based on DropletUtils::cleanTagCounts()."

8. Although the resource includes functionality to merge datasets, it lacks batch correction utilities. Given the diversity of data sources, either batch effects should be corrected directly or it should be demonstrated that they do not materially affect the data.

The reviewer is correct; scpca-nf does not include batch correction utilities.

The data available from the ScPCA Portal is designed to be flexible for users' many downstream applications, whether working with individual samples or multiple samples within a project. We do not intend to provide an atlas in the sense that all samples in ScPCA are integrated together, but rather a collection of single-cell profiles from a broad collection of pediatric cancer types. Aiming for flexibility is why, for example, multiple processing stages (unfiltered, filtered, and processed) are available from the Portal. This design philosophy underlies our choice not to include integrated or batch-corrected data in the Portal. We believe it is not possible for us to cover users' use cases without a substantive understanding of their intended analyses and experimental design (e.g., which covariates should be included in correction, whether diagnoses should be integrated separately).

Batch correction can also introduce artifacts (Antonsson and Melsted. 2025.). Many measures of the effectiveness of integration approaches (or detecting the presence of overcorrection) rely on high-quality cell type labels (Luecken et al. 2022.). As discussed throughout our response, we do not expect that our automated cell type annotations could identify high-quality cell type or state labels for malignant cells. In fact, in 2022-2023, we explored potentially adding integration to scpca-nf (as the reviewer can see here: https://github.com/AlexsLemonade/sc-data-integration) and determined that the absence of high-quality malignant cell labels was a significant barrier to reliable batch correction in this data.

In summary, we believe that providing integrated or batch-corrected data assets without a thorough performance assessment (including overcorrection) and a clear understanding of users' specific use cases may actively harm our users. This is because we may inadvertently introduce technical artifacts that impact users' experiments.

9. The manuscript focuses largely on describing the dataset and analysis workflow but provides minimal biological interpretation or translational insight. Including exemplar analyses of tumor, immune, and stromal compartments to identify biologically or clinically relevant features—such as outcome-associated signatures or novel cell states—would help demonstrate the resource's utility and better justify publication.

Our goal with the manuscript is to clearly describe what users of the ScPCA resource can obtain from the ScPCA Portal, explain the steps taken to prepare the data, and outline what users of our Nextflow workflow can expect. Although we have some preliminary findings we could share, we have taken great care to avoid competing with or "scooping" data generators/submitters in this manuscript. We firmly believe that data submitters/generators should have the opportunity

to publish on biologically or clinically relevant features, such as novel cell states or outcome-associated signatures, without our interference.

Regarding the resource's utility, we believe the Portal's usage demonstrates just that. Over 930 unique downloaders have used the ScPCA Portal, with more than 250 of which have been added since the manuscript's submission at the end of May. Since mid-December 2024, over 3700 downloads have been generated (as of January 29, 2026). The data provided by the ScPCA Portal have already been included in publications and preprints such as DeMartino et al. 2023., Mumme et al. 2023., Cao et al. 2025., Stöber et al. 2025., Zeng et al. 2025., Pushel et al. 2025., Elaskalani et al. 2025., and Budhathoki et al. 2026.

In this revision, we added text to the manuscript's introduction to demonstrate the resource's utility, which includes the number of unique downloaders, which is expected to quickly become outdated due to growth (lines 117 - 118):

"As of January 2026, over 900 unique downloaders have accessed the Portal since its launch."

Although this decision ultimately rests with the Editors and Reviewers, we respectfully believe that this meets the requirements for publication as a Resource article in Cell Genomics for "datasets [that] are openly accessible in public databases, meet FAIR guiding principles, provide a substantial advancement over prior resources, and are widely used to support the advancement of genomics and/or biomedical research," given the resource's open-access model, standardized formats and harmonized metadata, usage, and unique focus on pediatric cancer.

10, The manuscript repeatedly refers to the resource as "open source," but without raw FASTQ data and full metadata, this description is misleading. The scope of "open source" and any plans for raw data release should be clarified. Raw data must be made available

Most instances of the "open-source" language refer directly to the scpca-nf workflow, which is licensed under the BSD 3-clause license. The BSD 3-clause license is an open-source license approved by the Open Source Initiative (https://opensource.org/license/bsd-3-clause). Therefore, we believe the term "open-source" is appropriate for describing the workflow. The one instance in the introduction that used the phrase "an open-source data resource" was removed to avoid confusion, though the source code for the ScPCA Portal itself is identically openly licensed.

As stated in our response to the reviewer's 1st point, we have made as much metadata as possible available on the Portal and will not make raw, sequence-level data available, as it is sensitive human-subjects data, and this manuscript concerns an open-access resource. However, we wholeheartedly agree that raw data should be made available in widely used, government-run repositories that often allow access only when an appropriate Data Access Committee has approved it. We at Alex's Lemonade Stand Foundation cannot complete the institutional certification process for submission, e.g., to dbGaP, and therefore, rely on data generators/submitters to complete these steps. We are in contact with all researchers who received an ScPCA grant in 2019 to ensure deposition of raw data in repositories, and, in the majority of cases, deposition is in progress if not already completed.

When raw data is available in a government-run repository, as it is for 9 projects as of January 2026, the links to those accessions are present on the project's page to assist our users. Here is an example of a project with raw data deposited in dbGaP: https://scpca.alexslemonade.org/projects/SCPCP000003. We also retain submitter identifiers in

the metadata available in the Portal to facilitate the use of ScPCA data with other resources, such as dbGaP datasets. We will continue to add links to external accessions to projects as they become available.
We have added the following to the Results section (lines 167 - 169):
"Raw FASTQ files are not available for download from the Portal, but we direct users to where they can access the raw FASTQ files via links to external repositories, such as the Database of Genotypes and Phenotypes (dbGaP) (Mailman et al. 2007.; Tryka et al. 2014.), when available."
11. Figure 6 shows only broad cell lineages that can be reproduced by standard workflows. Including higher-resolution annotations (e.g., T cell subsets and tumor subpopulations) would make it more informative.
In Figure 6, which compares bulk and pseudobulk modalities, the displayed cell types are only those with marker genes that are significantly overrepresented among "positive outliers" (i.e., genes with higher expression in bulk RNA-seq than expected based on pseudobulk expression). If the reviewer is referring to Figure S6 (now Figure S5), we have included an additional dot plot of marker gene expression, shown in Figure 4D, for finer-grained immune subsets, as stated in our response to the reviewer's 5th point. If the reviewer refers to Figure 5 (now Figure 4), note that panel C does include myeloid and T cell subsets.
As suggested by Reviewer 2, we have added another cell type annotation method, SCimilarity, to scpca-nf. We have accordingly modified our consensus cell typing approach to label cells when two out of three methods (SingleR, CellAssign, and SCimilarity) agree. Including SCimilarity indeed yielded additional, finer consensus cell type labels.
As stated in our response to the reviewer's 5th and 6th points, we do not, and cannot, automatically classify tumor cells. We expect that many of the "unknown" cells shown in the figures mentioned above are, in fact, tumor cells. However, by adding InferCNV inference to the scpca-nf pipeline, as suggested by the reviewer, we can now provide additional cell-level information that researchers can use alongside consensus cell types to identify malignant cells. In addition, we have added trusted annotations from the OpenScPCA project to two projects (SCPCP000004 and SCPCP000015), which contain explicit malignant cell annotations, and we have included a new figure (now Figure 5) highlighting OpenScPCA labels for SCPCP000004.
12. Clarify whether the resource provides batch correction tools in addition to merging functionality, or state explicitly that users must handle corrections themselves.
As stated above in response to the reviewer's 4th point and expanded upon in response to the reviewer's 8th point, no batch correction is performed. As stated in our response to the reviewer's 4th point, we have added text to the manuscript explicitly stating that users must perform batch correction if their experimental design requires it.
Regarding users of the Portal itself, our documentation on merged objects (https://scpca.readthedocs.io/en/stable/processing_information.html#merged-objects) states, "These merged objects were not batch-corrected; they do not represent integrated objects."
We also include a warning in the download modal and the HTML report users receive alongside merged objects. We include examples on the next page.

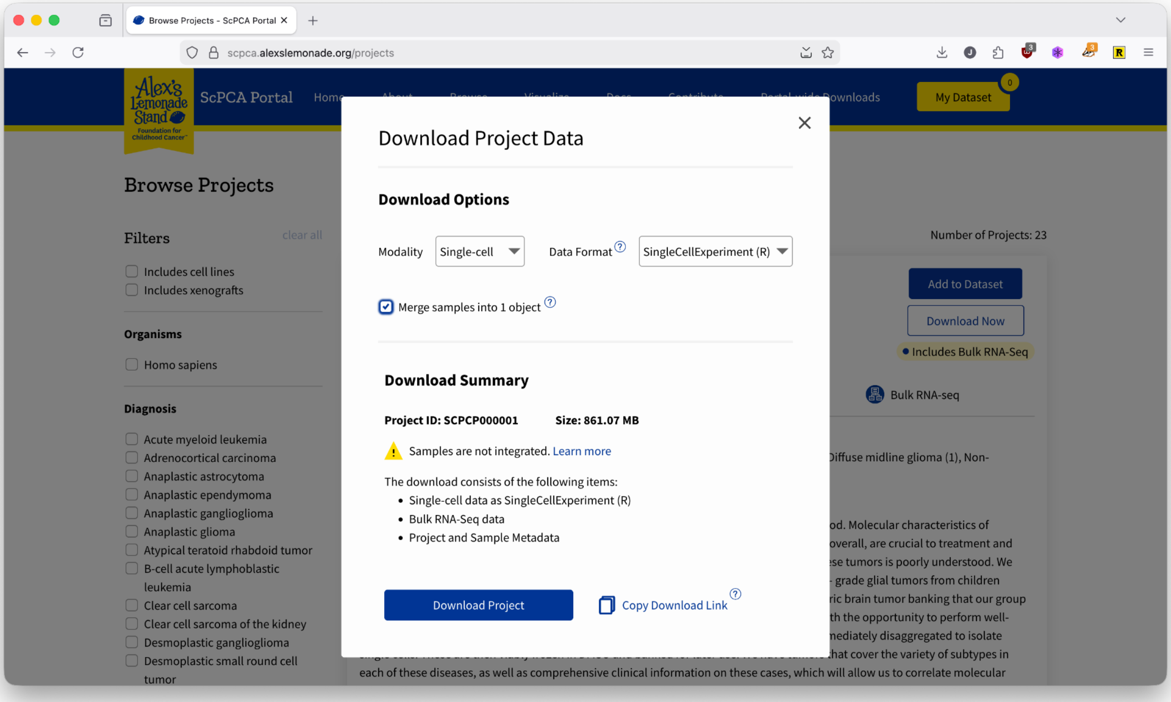
Browse Projects - ScPCA Portal
scpca.alexslemonade.org/projects
Alex's Lemonade Stand Foundation for Childhood Cancer
ScPCA Portal
My Dataset
Browse Projects
Filters
clear all
Includes cell lines
Includes xenografts
Organisms
Homo sapiens
Diagnosis
Acute myeloid leukemia
Adrenocortical carcinoma
Anaplastic astrocytoma
Anaplastic ependymoma
Anaplastic ganglioglioma
Anaplastic glioma
Atypical teratoid rhabdoid tumor
B-cell acute lymphoblastic leukemia
Clear cell sarcoma
Clear cell sarcoma of the kidney
Desmoplastic ganglioglioma
Desmoplastic small round cell tumor
Number of Projects: 23
Add to Dataset
Download Now
Includes Bulk RNA-Seq
Bulk RNA-seq
Download Project Data
Download Options
Modality
Single-cell
Data Format
SingleCellExperiment (R)
Merge samples into 1 object
Download Summary
Project ID: SCPCP000001
Size: 861.07 MB
Samples are not integrated. Learn more
The download consists of the following items:
Single-cell data as SingleCellExperiment (R)
Bulk RNA-Seq data
Project and Sample Metadata
Download Project
Copy Download Link
each of these diseases, as well as comprehensive clinical information on these cases, which will allow us to correlate molecular

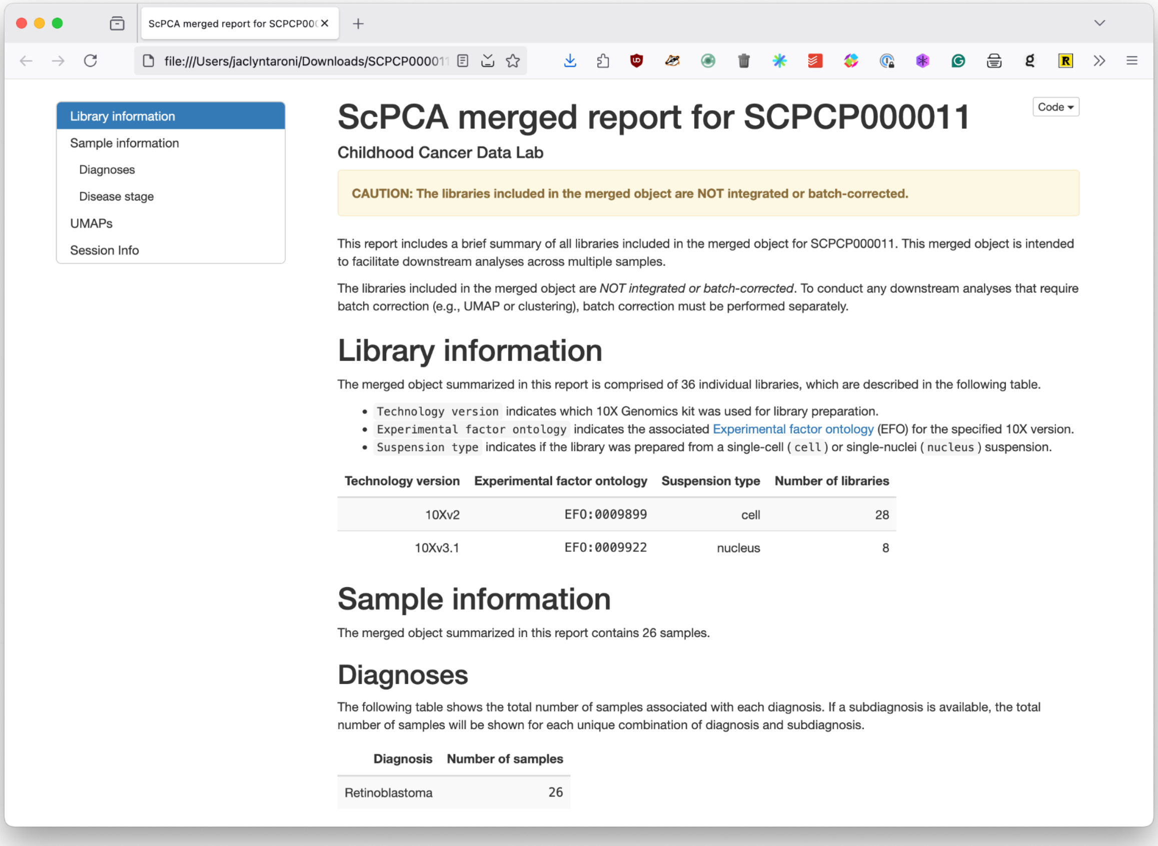
ScPCA merged report for SCPCP00
file:///Users/jaclyntaroni/Downloads/SCPCP00001
Library information
Sample information
Diagnoses
Disease stage
UMAPs
Session Info
Code
ScPCA merged report for SCPCP000011
Childhood Cancer Data Lab
CAUTION: The libraries included in the merged object are NOT integrated or batch-corrected.
This report includes a brief summary of all libraries included in the merged object for SCPCP000011. This merged object is intended to facilitate downstream analyses across multiple samples.
The libraries included in the merged object are NOT integrated or batch-corrected. To conduct any downstream analyses that require batch correction (e.g., UMAP or clustering), batch correction must be performed separately.
Library information
The merged object summarized in this report is comprised of 36 individual libraries, which are described in the following table.
Technology version indicates which 10X Genomics kit was used for library preparation.
Experimental factor ontology indicates the associated Experimental factor ontology (EFO) for the specified 10X version.
Suspension type indicates if the library was prepared from a single-cell (cell) or single-nuclei (nucleus) suspension.
Technology version Experimental factor ontology Suspension type Number of libraries
10Xv2 EFO:0009899 cell 28
10Xv3.1 EFO:0009922 nucleus 8
Sample information
The merged object summarized in this report contains 26 samples.
Diagnoses
The following table shows the total number of samples associated with each diagnosis. If a subdiagnosis is available, the total number of samples will be shown for each unique combination of diagnosis and subdiagnosis.
Diagnosis Number of samples
Retinoblastoma 26

13. Ensure that methods for doublet detection, ADT background subtraction, and normalization are described clearly in the methods section for transparency and reproducibility.

We thank the reviewer for this helpful comment. As described earlier in our response, we have now both included results doublet detection in ScPCA objects on the Portal, and we have updated the Methods section to include this additional language about each topic:

- Doublet detection (lines 529 - 534):

"These filtered objects additionally contain results from doublet detection performed with scDblFinder::scDblFinder() (Germain et al. 2021.), including each cell's predicted class ("singlet" or "doublet") as well as the associated doublet score. However, predicted doublets were not filtered out; users can instead use these scDblFinder results to filter doublets as needed for their specific analysis needs."

- ADT background subtraction (lines 590 - 593):

"Additionally, note that we did not perform background subtraction on the ADT counts, but we provide the ambient profile calculated with DropletUtils::ambientProfileEmpty(), which users can employ to perform global de-noising as needed."

- ADT normalization (lines 580 - 587; new text is shown in bold):

"ADT count data were then normalized using scuttle::computeMedianFactors(), which calculates a per-cell size factor as the median ratio of the cell's counts to the background profile previously calculated with DropletUtils::ambientProfileEmpty(). We then used these factors to normalize ADT counts with scuttle::logNormCounts(). If median-based normalization failed for any reason, ADT counts were log-transformed after adding a pseudocount of 1. We only performed normalization on cells that would be retained after ADT filtering; we assigned NA normalized counts to any cells that would be filtered out based on DropletUtils::cleanTagCounts()."

Overall, while this dataset has the potential to serve as a valuable community resource for pediatric tumor research, the current manuscript falls short in several critical areas, including data accessibility, usability, methodological transparency, and biological interpretation. Addressing these issues, by providing raw data and metadata, ensuring proper batch correction and annotation, improving user accessibility, and including analyses that yield meaningful biological or clinical insights would substantially enhance the impact and value of this work.

Referees' report, second round of review:

Reviewer #1: I would like to thank the authors for now including the UCSC browser versions of the data. There are a few suggested revisions that would greatly benefit the utility of this excellent resource

1. The overview tab list the 20+ browsers by SCPCP#. Am more descriptive name would be very helpful for quick navigation
2. Within each dataset there is a browser for each individual sample. Could the authors provide a master browser with all samples combined?
3. A list of marker genes pops up when moving the cursor over populations. Could the gene IDs be changed from ENSG# to gene symbol?

Reviewer #2: The authors addressed all my points and substantially improved their work by also providing an interactive cell browser for no-code browsing the data.

Authors' response to the second round of review

Reviewer #1: I would like to thank the authors for now including the UCSC browser versions of the data. There are a few suggested revisions that would greatly benefit the utility of this excellent resource

1. The overview tab list the 20+ browsers by SCPCP#. Am more descriptive name would be very helpful for quick navigation

Thank you for raising this important point. We have added the project title in addition to the SCPCP identifier to make browsing easier.

2. Within each dataset there is a browser for each individual sample. Could the authors provide a master browser with all samples combined?

Thank you for this suggestion. We do not make integrated objects available on the ScPCA Portal because we are concerned about introducing artifacts through the application of batch correction algorithms (Antonsson and Melsted. 2025.). Instead, we leave the batch correction approach that is best suited to a given analysis up to the user. Including a master browser with all samples that are merged but unintegrated may be misleading and is incongruous with the design of the ScPCA Portal data downloads. If integrated data ever become available on the ScPCA Portal, we will revisit this suggestion.

3. A list of marker genes pops up when moving the cursor over populations. Could the gene IDs be changed from ENSG# to gene symbol?

Thank you for this recommendation. We have updated how the annotations for marker genes are populated on our Cell Browser instance so the marker gene list is now displayed by gene symbols. Clicking on the popup opens a table with both Ensembl gene ids and gene symbols, along with some additional annotation and links to external resources.
